# Treatment With a Marine Oil Supplement Alters Lipid Mediators and Leukocyte Phenotype in Healthy Patients and Those With Peripheral Artery Disease

**DOI:** 10.1161/JAHA.120.016113

**Published:** 2020-07-22

**Authors:** Melinda S. Schaller, Mian Chen, Romain A. Colas, Thomas A. Sorrentino, Ann A. Lazar, S. Marlene Grenon, Jesmond Dalli, Michael S. Conte

**Affiliations:** ^1^ Division of Vascular and Endovascular Surgery Cardiovascular Research Institute University of California, San Francisco San Francisco CA; ^2^ William Harvey Research Institute Barts and The London School of Medicine and Dentistry Queen Mary University of London London United Kingdom; ^3^ Department of Epidemiology and Biostatistics University of California, San Francisco San Francisco CA; ^4^ Centre for Inflammation and Therapeutic Innovation Queen Mary University of London London United Kingdom

**Keywords:** fatty acids, inflammation, lipid metabolites, peripheral artery disease, vascular disease, Peripheral Vascular Disease, Vascular Disease, Clinical Studies, Inflammation, Lipids and Cholesterol

## Abstract

**Background:**

Peripheral artery disease (PAD) is an advanced form of atherosclerosis characterized by chronic inflammation. Resolution of inflammation is a highly coordinated process driven by specialized pro‐resolving lipid mediators endogenously derived from omega‐3 fatty acids. We investigated the impact of a short‐course, oral, enriched marine oil supplement on leukocyte phenotype and biochemical mediators in patients with symptomatic PAD and healthy volunteers.

**Methods and Results:**

This was a prospective, open‐label study of 5‐day oral administration of an enriched marine oil supplement, assessing 3 escalating doses in 10 healthy volunteers and 10 patients with PAD. Over the course of the study, there was a significant increase in the plasma level of several lipid mediator families, total specialized pro‐resolving lipid mediators, and specialized pro‐resolving lipid mediator:prostaglandin ratio. Supplementation was associated with an increase in phagocytic activity of peripheral blood monocytes and neutrophils. Circulating monocyte phenotyping demonstrated reduced expression of multiple proinflammatory markers (cluster of differentiation 18, 163, 54, and 36, and chemokine receptor 2). Similarly, transcriptional profiling of monocyte‐derived macrophages displayed polarization toward a reparative phenotype postsupplementation. The most notable cellular and biochemical changes over the study occurred in patients with PAD. There were strong correlations between integrated biochemical measures of lipid mediators (specialized pro‐resolving lipid mediators:prostaglandin ratio) and phenotypic changes in circulating leukocytes in both healthy individuals and patients with PAD.

**Conclusions:**

These data suggest that short‐term enriched marine oil supplementation dramatically remodels downstream lipid mediator pathways and induces a less inflammatory and more pro‐resolution phenotype in circulating leukocytes and monocyte‐derived macrophages. Further studies are required to determine the potential clinical relevance of these findings in patients with PAD.

**Registration:**

URL: https://www.clinicaltrials.gov; Unique identifier: NCT02719665.

**Nonstandard Abbreviations and Acronyms**
17‐HDHA: 17‐hydroxy docosahexaenoic acid
AA: arachidonic acid
CD: cluster of differentiation
DHA: docosahexaenoic acid
DPA: docosapentaenoic acid
EPA: eicosapentaenoic acid
hs‐CRP: high‐sensitivity C‐reactive protein
IL: interleukin
LTB4: leukotriene B4
MCTR: maresin conjugate in tissue regeneration
MDM: monocyte‐derived macrophage
MFI: mean fluorescence intensity
n‐3: omega‐3
PAD: peripheral artery disease
PCTR: protectin conjugate in tissue regeneration
PLS‐DA: partial least squares discriminant analysis
PUFA: polyunsaturated fatty acid
RBC: red blood cell
RvD: D‐series resolvin
RvE: E‐series resolvin
RvT: 13‐series resolvin
SPM: specialized pro‐resolving lipid mediator

**Clinical Perspective**

**What Is New?**

- This is the first study to elucidate the omega‐3 and omega‐6 polyunsaturated fatty acid lipid mediator profile in peripheral artery disease.
- We have shown that a short‐course, oral marine oil supplement can remodel these lipid mediator pathways to induce a less inflammatory and pro‐resolution phenotype.

**What Are the Clinical Implications?**

- This study provides a foundation for characterizing biochemical and cellular biomarkers of inflammation and resolution in peripheral artery disease to allow for future work correlating upstream nutritional or pharmacologic interventions, immune cell function, and downstream clinical events.

Atherosclerosis is the leading cause of death worldwide. Peripheral artery disease (PAD), one of the most advanced forms of atherosclerosis, is estimated to affect 8.5 million individuals in the United States alone.^1^ With increased aging and risk factors such as diabetes mellitus, the prevalence of PAD is increasing around the globe, with a rise of ≈25% over the preceding decade.^2^ Atherosclerotic plaque is the result of a series of highly specific cellular and molecular responses that can best be described, in aggregate, as a chronic inflammatory disease.^3^, ^4^, ^5^, ^6^, ^7^, ^8^, ^9^, ^10^, ^11^, ^12^ This disease state begins from an inflammatory lesion, known as the fatty streak, which forms from peripheral blood mononuclear cells such as monocytes, and can progress to occlusive lesions causing significant morbidity, limb loss, and death.^12^

Uncontrolled or excessive inflammation, such as that seen in PAD, is associated with many chronic disease processes. Recent evidence demonstrates that the resolution of inflammation occurs via a highly coordinated effort of active mediators and cellular processes.^13^ Many of the key mediators of resolution are derived from essential polyunsaturated fatty acid (PUFA) precursors and include resolvins, protectins, maresins, and lipoxins.^14^ These specialized pro‐resolving lipid mediators (SPMs) are produced endogenously from biochemical precursors via lipoxygenase and cyclo‐oxygenase enzymatic pathways, and engage specific cell surface receptors to mediate their downstream actions.^13^ Cardinal signs of resolution (SPM actions) include cessation of neutrophil accumulation, phagocytosis of necrotic cells and debris, efferocytosis, and tissue remodeling.^13^, ^15^ Importantly, the PUFA precursors of SPMs are poorly synthesized in mammals and must be derived from dietary sources such as marine oils.

Prior work has demonstrated that impaired resolution contributes to the progression of atherosclerotic cardiovascular disease in humans.^16^, ^17^, ^18^ Supplementation with omega‐3 (n‐3) PUFA has been shown to positively impact systemic SPM production as well as receptor expression.^19^, ^20^ Early clinical trials with n‐3 PUFA in cardiovascular disease yielded mixed results; however, more recent studies have demonstrated cardiac risk reduction associated with their use.^21^, ^22^, ^23^, ^24^ To this point, the dosing and formulations of n‐3 PUFA employed in clinical studies have varied, and surrogate biochemical or cellular biomarkers have not been established. Additionally, it is unknown whether SPM‐related biochemical pathways may be effectively targeted by nutritional supplementation to promote resolution. We aimed to investigate the impact of a short‐course, oral, enriched marine oil supplement on circulating leukocytes and biochemical mediators in patients with symptomatic PAD and healthy controls. This pilot study serves as a framework for future investigations of n‐3 PUFA supplements targeting resolution pathways, with an ultimate goal of developing new approaches to reduce disease progression and morbidity in patients with PAD.

## Methods

### Overview

**Figure 1:**
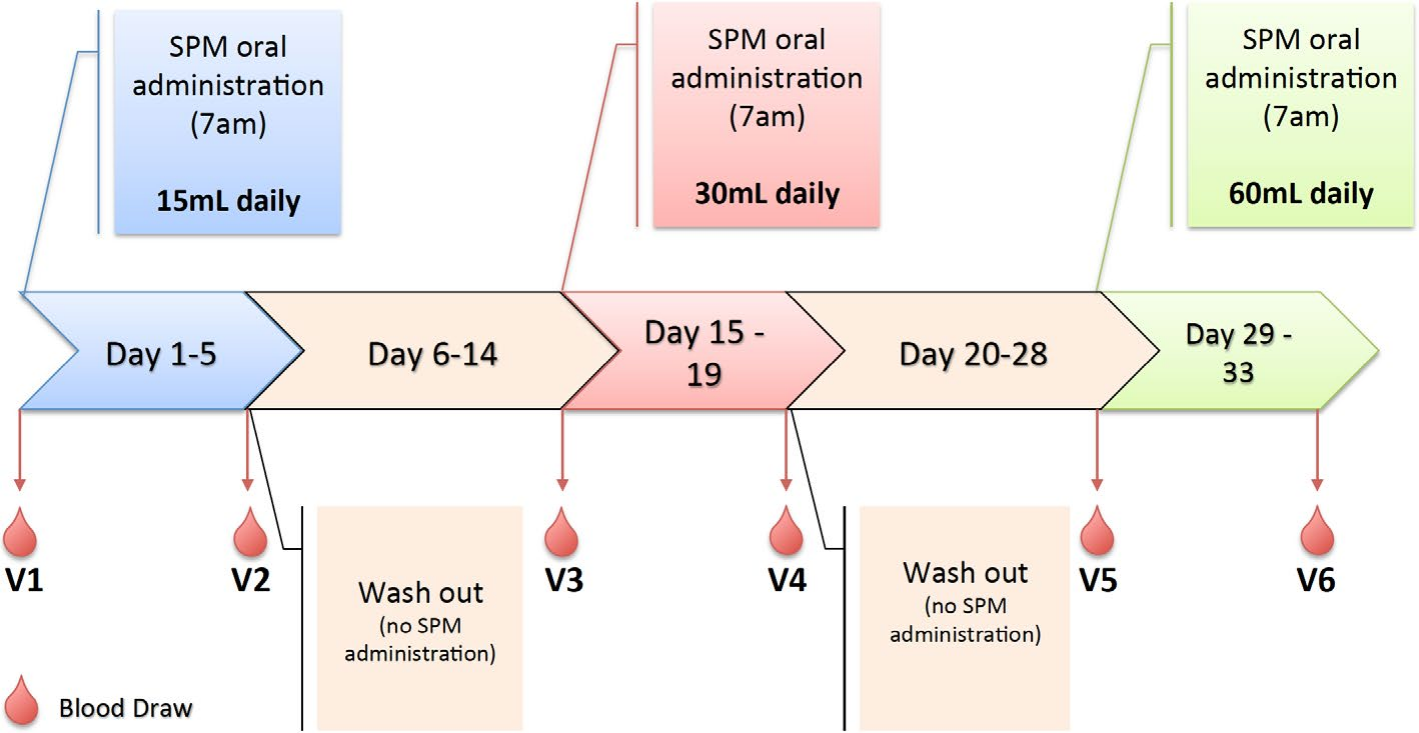
**Schematic of study design.** SPM indicates specialized pro‐resolving lipid mediator; and V, visit number.

The data that support the findings of this study are available from the corresponding author upon reasonable request. The OMEGA‐SPM‐DOSE trial was a pilot study that investigated the effects of an enriched marine oil supplement containing highly concentrated n‐3 PUFA and their metabolites on plasma lipid mediator profile, biomarkers of inflammation and resolution, and leukocyte phenotype in healthy individuals and patients with PAD. The primary goals of this investigation were to: (1) determine whether short‐term oral administration of an enriched marine oil supplement alters the plasma lipid mediator profiles of healthy volunteers and patients with PAD, and (2) define a dosing regimen to maximize SPM bioavailability. Secondary goals were to determine the effects of supplementation on inflammatory biomarkers, circulating leukocyte phenotype, bacterial phagocytosis activity, and monocyte‐derived macrophage (MDM) gene expression. The trial was accomplished as an investigator‐initiated study at a single center (University of California, San Francisco [UCSF]). This was a prospective, open‐label, nonblinded study of acute oral administration of an enriched marine oil supplement, assessing 3 escalating doses (15 mL, 30 mL, and 60 mL daily; corresponding to 1.5 g, 3.0 g, and 6.0 g of the enriched marine lipid oil), in 10 healthy volunteers and 10 patients with stable, symptomatic PAD. The study supplement was administered once daily for 5 days, followed by a 9‐day washout period, for a total of 33 days of study duration (Figure 1).

### Study Population

Healthy volunteers were recruited using posted advertisements. Once a potential participant initiated contact, they were screened for study eligibility. For healthy individuals, enrollment criteria included having an hs‐CRP (high‐sensitivity C‐reactive protein) <2 mg/L; those taking aspirin or nonsteroidal anti‐inflammatory drugs regularly were also excluded. Patients with PAD were recruited from the vascular surgery clinics at UCSF after being screened for eligibility (Table S1). Before participating in the study, all participants provided informed consent. Institutional review board approval was granted for this study by the Committee on Human Research at UCSF. The study was registered with the ClinicalTrials.gov (OMEGA‐SPM studies; identifier NCT02719665).

### Intervention

The enriched marine oil supplement test product was provided by Metagenics, Inc. It contained ≈46% eicosapentaenoic acid (EPA), ≈33% docosahexaenoic acid (DHA), and ≈18% n‐3 docosapentaenoic acid (DPA), as well as monohydroxylated SPM precursors such as 17‐hydroxy DHA (17‐HDHA) (500 μg/dL) and 18‐hydroxy EPA (287 μg/dL) (Solutex) (Table S2). The enriched marine lipid fraction used in this study is standardized to the SPM precursors 17‐HDHA and 18‐hydroxy EPA in addition to the esterified and free fatty acid concentrations. The study supplement was supplied as an emulsion in dose‐specific packages by the manufacturer to the research team. Study supplement packages were distributed at the beginning of each dosing period and contained the exact volume of the supplement for treatment for 5 days as appropriate to the incremental dose. The doses tested were 15 mL, 30 mL, or 60 mL per day for 5 days. This corresponds to ≈1.5 g, 3.0 g, and 6.0 g of enriched marine oil, per day, respectively. Participants were directed to take the specified dosage once daily orally. Optimal timing of the dose was in the morning with breakfast. Participants were instructed to continue to eat their typical diet but to avoid eating high‐fat foods with the supplement. No adjustments for weight or age were made. Each treatment period was 5 days, with a 9‐day washout between doses. Blood draws were conducted at the beginning and end of each treatment period. Samples were always collected at the same time of day, ≈4 hours after the last dose. Participants were instructed to fast for 4 hours, or after consuming the supplement, on the day of the blood draw.

### Primary and Secondary End Points

The primary end point was the change in the total plasma SPM level before and after each dosing interval. Total SPM was calculated as a sum of the concentration of the bioactive metabolomes of EPA, DHA, and n‐3 DPA, with the addition of lipoxins (see below). Secondary end points included the plasma ratio of total SPM to prostaglandins, a change in n‐3 index, selected inflammatory and resolution cytokines, monocyte and neutrophil phagocytosis of bacteria, circulating monocyte surface markers, and MDM gene expression.

### Demographics and Medical History

Demographic data, cardiovascular history, risk factors for PAD, medications, and examination findings pertinent to cardiovascular history were recorded.

### Taste and Tolerability

Participants were given worksheets at the beginning of each dosing period and were instructed to record the time they took the supplement, what they ate with it, and comments regarding their experience taking the supplement. These worksheets were collected and reviewed to confirm compliance.

### Blood Sample Collection

Blood samples were collected from participants at baseline (visit 1 [V1]) and before and after (4 hours after the final dose [V2–V6]) each treatment period and were processed immediately. Samples collected for serum inflammatory marker analysis were collected in serum‐separator tubes and allowed to clot for 1 hour at room temperature. Samples collected for plasma lipid mediator profiling were collected in EDTA tubes. Samples collected for red blood cell (RBC) analysis of the n‐3 index were collected in EDTA tubes. Serum, plasma, and the RBC pack were immediately frozen at −80°C. Samples collected for neutrophil, monocyte, and macrophage studies were collected in EDTA and heparin tubes and placed on ice before analysis.

### Monocyte and Neutrophil Phagocytosis by Flow Cytometry

Whole blood samples were obtained in heparin tubes. Blood samples were incubated with pHrodo *E. coli* (0.005 μg, Invitrogen) for 1 hour at 37°C or on ice. To each tube, Fc Block (15 μL, eBioscience) and cluster of differentiation (CD) 86 (5 μL, BioLegend) were added. CD86 was used to differentiate monocytes from other cell types, such as natural killer cells. Tubes were then incubated in the dark, on ice for 30 minutes with gentle agitation. The tubes were then treated with 1‐step Fix/Lyse Solution (eBioscience) and incubated for an additional 15 minutes at room temperature. Samples were then centrifuged at 500*g* for 5 minutes, washed with flow buffer, centrifuged again at 500*g* for 5 minutes, resuspended, and analyzed by flow cytometry (250 000 events per sample; BD FACSVerse, Becton, Dickinson and Company). Analysis was conducted using FlowJo software (FlowJo, LLC).

### Monocyte Cell Surface Markers

Whole blood samples were obtained in EDTA tubes. Blood was divided into aliquots and treated with Fc Block, Live/Dead (Invitrogen) and the following antibodies (5 μL per sample): CD14, CD16, CD86, CD54, CD163, CD18, CD36, and CD49d (BioLegend). Samples were incubated in the dark, on ice, for 30 minutes with gentle agitation. The tubes were then treated with 1‐step Fix/Lyse Solution (eBioscience) and incubated for an additional 15 minutes at room temperature. Samples were then centrifuged at 500*g* for 5 minutes, washed with flow buffer, centrifuged again at 500*g* for 5 minutes, resuspended, and analyzed by flow cytometry (250 000 events per sample, BD FACSVerse, Becton, Dickinson and Company). Analysis was conducted using FlowJo software (FlowJo, LLC). Quantitative data are described by mean fluorescence intensity (MFI) for each marker.

### Peripheral Blood Mononuclear Cell Isolation and MDM Differentiation

Peripheral blood mononuclear cells were isolated using SepMate tubes (S Technologies) according to protocol. Peripheral blood mononuclear cells were plated in 6‐well plates (Genesee Scientific) at a density of 3 million cells per well. Monocytes were isolated via adherence to the wells after a 2‐hour incubation. Plates were rinsed with PBS (Gibco) to remove nonadherent cells. Monocytes were then cultured for 1 week in RPMI (Gibco) with 5% heat inactivated FBS (Gibco) and 10 nmol/L GM‐CSF (R&D Systems) to allow for differentiation into macrophages (MDM).

### MDM Gene Expression Analysis

After a week in culture, MDMs were stimulated with lipopolysaccharide (10 ng/mL, Sigma‐Aldrich) to mimic the occurrence of an acute inflammatory event, or vehicle control for 24 hours before assessment of gene expression. MDM gene expression was assessed at 2 study time points: before beginning supplementation (V1) and after completing the highest dose of the supplement (V6). Cells were then lysed using RLY buffer (Bioline) and the lysate frozen at −80°C until analysis. RNA was isolated according to protocol (Qiagen). Reverse transcription was conducted per protocol using a High‐Capacity cDNA Reverse Transcription Kit (Applied Biosystems) and PCR was performed via incorporation of SYBR Green (Applied Biosystems) using a CFX86 RT PCR System (Bio‐Rad). Analyses were conducted using Bio‐Rad CFX Analysis software (Bio‐Rad) and were normalized to the housekeeping gene HPRT.

### n‐3 Index

Packed RBCs were stored at −80°C until batch assayed for n‐3 PUFA content of EPA and DHA, the n‐3 index (OmegaQuant).^25^, ^26^ This analysis was blinded to patient type (healthy versus PAD) and visit number.

### Targeted Lipid Mediator Profiling

Targeted lipid mediator profiling was performed on plasma samples using liquid chromatography‐tandem mass spectrometry. Plasma levels of the free fatty acids EPA, DHA, n‐3 DPA, and arachidonic acid (AA) were also measured. Within each of the specific lipid mediator families, the precursors and pathway markers, as well as the bioactive mediators themselves, were measured.^27^ The DHA bioactive metabolome includes the D‐series resolvins (RvD1, RvD2, RvD3, RvD4, RvD5, RvD6, 17R‐RvD1, and 17R‐RvD3), protectins (PD1, 10S,17S‐diHDHA, 17R‐PD1, and 22‐OH‐PD1), protectin conjugates in tissue regeneration (PCTR1, PCTR2, and PCTR3), maresins (MaR1, 7S, 14S‐diHDHA, MaR2, 4S, 14S‐diHDHA, and 22‐OH‐MaR1), and maresin conjugates in tissue regeneration (MCTR1, MCTR2, and MCTR3). The n‐3 DPA bioactive metabolome includes the n‐3 DPA‐derived 13‐series resolvins (RvT1, RvT2, RvT3, and RvT4), RvDs (RvD1_n‐3 DPA_, RvD2_n‐3 DPA_, and RvD5_n‐3 DPA_), maresins (MaR1_n‐3 DPA_ and 7S, 14S‐diHDPA), and protectins (PD1_n‐3 DPA_ and 10S, 17S‐diHDPA). The EPA bioactive metabolome includes the E‐series resolvins (RvE1, RvE2, and RvE3). The AA bioactive metabolome includes lipoxins (LXA_4_, LXB_4_, 5S, 15S‐diHETE, 15‐epi‐LXA_4_, and 15‐epi‐LXB_4_), AA‐derived leukotrienes (LTB_4_, 5S, 12S‐diHETE, 12‐epi‐LTB_4_, 6‐trans, 12‐epi‐LTB_4_, and 20‐OH‐LTB_4_), cysteinyl leukotrienes (LTC_4_, LTD_4_, and LTE_4_), prostaglandins (PGD_2_, PGE_2_, and PGF_2a_), and thromboxane. Total SPM was calculated as a sum of the concentration of the bioactive metabolomes of DHA, EPA, and n‐3 DPA with the addition of lipoxins. This analysis was blinded to patient type (healthy versus PAD) and visit number.

### Serum Inflammatory Markers

Serum was stored at −80°C until assayed for hs‐CRP, total adiponectin, monocyte chemoattractant protein‐1, and interleukin (IL) 6 by the CERLab at Boston Children's Hospital (Boston, MA). This analysis was blinded to patient type (healthy versus PAD) and visit number.

### Statistical Analysis

Statistical analyses and data derivation were performed in Stata (version 13.0, StataCorp), SAS (version 9.4; SAS Institute Inc), and Microsoft Excel. Baseline characteristics of the study participants are displayed as median (interquartile range) or mean (SD or SEM) for continuous data and number (percentage) for categorical data. Paired or unpaired Student *t* tests and nonparametric, Wilcoxon signed rank (paired test), and Mann–Whitney tests were performed as appropriate for within‐group or between‐group comparisons, respectively. Chi‐square and Fisher exact tests were performed for categorical variables. The normality test was used to determine which test (parametric or nonparametric) was appropriate.

To address the question of which dose of the supplement led to the greatest impact on the outcomes of interest, a linear mixed model (y_ij_=β_o_+visit_j_+patient_j_+ε_ij_ with ε_ij_ ~ i.i.d $N(0,\sigma_{\varepsilon }^{2})$ and patient_j_ $∼N(0,\sigma_{\text{subject}}^{2})$ independently of ε_ij_ of the specified outcome y_ij_ for person i with visit j where j=1–6 visits) with random intercept fit to this longitudinal data to account for the correlation among patients measured across multiple visits.^28^ A linear mixed model with random intercepts was also generated to assess the association between biological outcomes (eg, phagocytic activity and cell surface marker expression) and biochemical predictors (total SPM, total SPM:prostaglandin ratio). For this model, we fit an interaction between visit and biological predictor (y_ij_=visit_j_+biological predictor_j_×visit_j_+patient_j_+ε_ij_). We investigated whether quadratic effects improved the model fit using a likelihood ratio test (*P*<0.05). We did not find evidence of this for any of the models evaluated. No additional covariates were adjusted for in any of the models. Kenward‐Roger denominator degrees of freedom were used to make small sample inference.^29^ Three separate models were assessed for each outcome based on the: (1) entire cohort, (2) healthy subgroup, and (3) PAD subgroup. We estimated differences from baseline at each dose and across doses. We assessed the standardized residuals with respect to normal quantiles using a QQ plot, and log‐transformed end points as appropriate. Since the results in terms of statistical significance for the log‐transformed end points were similar, the untransformed results are presented for ease of interpretation. Two‐sided *P*<0.05 were considered statistically significant. We assessed carryover effects in each cohort between visits 1, 3, and 5 by using an F test (contrast statement in SAS proc mixed).

To assess differences in lipid mediator profiles between baseline and the different supplement groups we used partial least squares discriminant analysis (PLS‐DA) that was performed using the MetaboAnalyst statistical analysis tool.^30^ Here, features with a constant or single value across samples were deleted. PLS‐DA was then performed following auto‐scaling (mean‐centered and divided by the SD of each variable). PLS‐DA is based on a linear multivariate model that identifies variables that contribute to class separation of observations on the basis of their variables (lipid mediator levels). During classification, observations were projected onto their respective class model. The score plot illustrates the systematic clusters among the observations (closer plots presenting higher similarity in the data matrix).

## RESULTS

### Patient Demographics

Twenty patients completed the study: 10 with PAD, defined as those with mild to severe claudication with a resting ankle brachial index <0.9 or toe brachial index <0.6, and 10 healthy individuals, defined as those without any chronic diseases and an hs‐CRP <2.0 mg/L. A summary of the baseline characteristics of this cohort is included in Table 1.

**Table 1:** Baseline Patient Demographics

Characteristic	Healthy Participants (n=10)	Patients With PAD (n=10)	*P* Value
Age, median (IQR), y	54.4 (32, 60)	69.5 (61, 75)	0.001*
Men	4 (40.0)	7 (70.0)	0.196
Body mass index, mean (SD)	25.5 (3.8)	26.5 (5.4)	0.637
Race/ethnicity			0.632
White	7 (70.0)	6 (60.0)	
Black	1 (10.0)	2 (20.0)	
Asian	1 (10.0)	0	
Hispanic/Latino	1 (10.0)	2 (20.0)	
Clinical measurements at baseline			
White blood cell count, mean (SD)	5.82 (1.37)	7.51 (1.44)	0.015*
Hemoglobin, mean (SD)	13.9 (1.09)	13.7 (1.52)	0.715
Hematocrit, mean (SD)	41.5 (2.11)	41.6 (4.87)	0.958
Platelet count, median (IQR)	267 (197–280)	241 (193–317)	0.496
Creatinine, mean (SD)	0.88 (0.21)	1.04 (0.19)	0.093
n‐3 index, mean (SEM)	5.7 (0.2)	4.9 (0.5)	0.041*
hs‐CRP, median (IQR)	0.5 (0.3–0.9)	4.5 (1.9–6.2)	0.001*
Medical history			
Stroke	0	0	
Coronary artery disease	0	2 (20.0)	0.151
Congestive heart failure	0	0	
Hypertension	0	8 (80.0)	0.001*
Hyperlipidemia	0	9 (90.0)	<0.001*
Diabetes mellitus	0	2 (20.0)	0.151
Type 1		0 (0)	
Type 2		2 (100.0)	
History of tobacco use	4 (40.0)	9 (90.0)	0.018*
Daily aspirin use	0	9 (90.0)	<0.001*
Daily clopidogrel use	0	6 (60.0)	0.002*
Daily statin use	0	7 (70.0)	0.003*

Values are expressed as number (percentage) unless otherwise indicated. hs‐CRP indicates high‐sensitivity C‐reactive protein; IQR, interquartile range; n‐3, omega‐3; and PAD, peripheral artery disease.

* *P*<0.05.

No adverse reactions occurred during the study. Two participants dropped out of the study (9%) (Figure S1). One participant dropped out secondary to the development of a gout flare before starting the supplement. A second participant decided the time commitment was too great. Taste and tolerability data were collected from all patients. Seven patients reported negative comments regarding their experience taking the supplement, including bad taste and burping. Compliance with taking the supplement was 100% among all participants.

### n‐3 Index

The n‐3 index is a validated biomarker used to define the RBC content of EPA and DHA, thus it reflects the interplay between oral intake and metabolism of n‐3 and omega‐6 PUFA.^25^, ^26^ Identifying the percentage contribution of EPA and DHA to total RBC fatty acids accurately reflects plasma and tissue levels of EPA and DHA, and may be considered a measure of compliance with the study protocol. At baseline, the n‐3 index was significantly lower among patients with PAD compared with healthy volunteers (PAD 4.9±0.3 versus healthy 5.7±0.2, *P*=0.041). Over the course of the study, an increase in the n‐3 index was observed in both the patients with PAD (4.9±0.3 [V1] to 6.5±0.3 [V6], *P*<0.001) and the healthy individuals (5.7±0.2 [V1] to 6.9±0.2 [V6], *P*<0.001). Changes in the n‐3 index were dose‐dependent, as expected (Figure S2). There was a statistically significant carryover effect in the n‐3 index between doses, as the subsequent presupplement visits (V3 and V5) did not return to study baseline (V1).

### Serum Inflammatory Markers

Selected inflammatory markers were measured in each participant at all time points. At baseline, both hs‐CRP and IL‐6 were significantly higher in patients with PAD (hs‐CRP: PAD 3.7±0.9 versus healthy 0.6±0.1 [*P*=0.004]; IL‐6: PAD 5.1±1.4 versus healthy 1.1±0.2 [*P*=0.011]). Over the course of the study, both hs‐CRP and IL‐6 levels trended downward. While not reaching statistical significance, these trends were largely driven by the PAD cohort, in whom the hs‐CRP level decreased from 3.7±0.93 to 3.0±0.6 mg/L (*P*=0.325) and the IL‐6 level decreased from 5.1±1.4 to 3.8±0.8 pg/mL (*P*=0.147). There was no change in the inflammatory markers in the healthy cohort.

### Phagocytic Ability of Monocytes and Neutrophils

We characterized the phagocytic ability of circulating monocytes and neutrophils (polymorphonuclear neutrophils) throughout the study period (Table S3). At V1, there was no significant difference in circulating leukocyte phagocytosis activity between patients with PAD and healthy participants. Compared with baseline, monocyte phagocytosis of labeled *E. coli* increased (normalized MFI 58±3.7 [V1] to 64±2.9 [V6], *P*=0.014) with supplementation within the PAD cohort. There was no significant change in monocyte phagocytosis within the healthy cohort (MFI 55±1.3 [V1] to 58±2.9 [V6], *P*=0.357). Among the healthy participants, neutrophil phagocytosis increased (MFI 63±2.7 [V1] to 75±3.8 [V6], *P*=0.002) following supplementation. There was also a trend toward increased neutrophil phagocytosis in the PAD cohort, although this did not reach statistical significance (MFI 66±5.2 [V1] to 73±5.8 [V6], *P*=0.176).

### Monocyte Cell Surface Markers

The phenotype of circulating monocytes was investigated by cell surface marker expression via flow cytometry. There was no significant difference in monocyte cell surface marker expression between the healthy participants and the patients with PAD at baseline (V1). Within the PAD cohort, we observed decreased expression of the monocyte adhesion molecule CD18 (MFI 6403±468 [V1] to 5264±380 [V6], *P*<0.001) expressed on activated monocytes, intercellular adhesion molecule 1 (MFI 3505±198 [V1] to 3083±217 [V6], *P*=0.003) and chemokine receptor 2 (MFI 4998±317 [V1] to 4377±318 [V6], *P*=0.006), both involved with leukocyte infiltration, and the scavenger receptors CD163 (MFI 2971±157 [V1] to 2444±136 [V6], *P*=0.003) and CD36 (MFI 24 255±1822 [V1] to 20 396±1211 [V6], *P*=0.001), involved in chronic inflammation and the uptake of oxidized low‐density lipoproteins, respectively (Table 2). Within the healthy cohort, only CD18 (MFI 6837±370 [V1] to 5522±288 [V6], *P*=0.007) and CD36 (MFI 21 266±1689 [V1] to 19 468±1307 [V6], *P*=0.041) significantly decreased (Table 2).

**Table 2:** Monocyte Surface Marker Expression Before and After Supplementation

Cell Surface Marker	Healthy Participants			Patients With PAD		
	Study Start (V1)	Study End (V6)	*P* Value	Study Start (V1)	Study End (V6)	*P* Value
CD18	6837±370	5522±288	0.007*	6403±468	5264±380	<0.001*
CD163	2920±240	2585±160	0.076	2971±157	2444±136	0.003*
CD54/ICAM‐1	3234±150	3250±206	0.864	3505±198	3083±217	0.003*
CCR2	4277±317	4547±284	0.208	4998±317	4377±318	0.006*
CD49d	2498±187	2592±195	0.291	2118±240	2122±270	0.978
CD36	21 266±1689	19 468±1307	0.041*	24 255±1822	20 396±1211	0.001*

Values are median fluorescence intensity (MFI) and expressed as mean±SEM. CCR2 indicates chemokine receptor 2; CD, cluster of differentiation; ICAM‐1, intercellular adhesion molecule 1; PAD, peripheral artery disease; and V, visit number.

* *P*<0.05 by paired Student *t* test (within group).

### MDM Gene Expression

Macrophage phenotype plays a critical role in the local inflammatory response and resolution of tissue injury. Type 1 macrophages are associated with inflammatory states and type 2 macrophages are associated with the resolution of inflammation and tissue repair.^31^ Human macrophages cannot be directly isolated from blood; therefore, we differentiated peripheral blood monocytes into MDM in culture.^32^ After a week in culture, we stimulated MDM with lipopolysaccharide, to mimic an acute inflammatory event, or vehicle for 24 hours before assessment of gene expression. MDM gene expression was assessed before beginning supplementation and after completing the highest dose of the supplement. Baseline expression of target genes was compared between the PAD cohort and the healthy cohort. MDM from patients with PAD at baseline (V1) demonstrated significantly increased expression of tumor necrosis factor‐α, monocyte chemoattractant protein‐1, C‐X‐C motif chemokine 10, and IL‐10, and reduced expression of chemokine C‐C motif ligand 17, and mannose receptor C type 1 compared with healthy participants, consistent with a greater type 1 macrophage versus type 2 macrophage polarization in patients with PAD (Table S4).

To investigate changes in MDM gene expression after marine oil supplementation, the fold change in the target gene at the final time point (V6), normalized to the housekeeping gene HPRT and relative to the expression at the baseline time point (V1), was calculated for each sample (Table 3). Within the PAD cohort, a decrease in MDM gene expression of monocyte chemoattractant protein‐1 (expression fold change, 0.51±0.10; *P*=0.008), inducible nitric oxide synthase (expression fold change, 0.68±0.09; *P*=0.023), and C‐X‐C motif chemokine 10 (expression fold change, 0.25±0.06; *P*=0.043), all associated with the type 1 macrophage phenotype, occurred over the course of treatment. In contrast, MDM expression of the mannose receptor C type 1 gene, a type 2 macrophage marker, was upregulated in the PAD cohort (expression fold change, 2.49±1.10; *P*=0.040). In the PAD cohort, treatment with the supplement additionally resulted in decreased IL‐10 gene expression (expression fold change, 0.39±0.06; *P*=0.001) by MDM following lipopolysaccharide stimulation. There was no statistically significant change in any of the MDM gene expression over the study period within the healthy cohort.

**Table 3:** Changes in MDM Gene Expression After Supplementation

Gene	M1 or M2	Healthy Participants		Patients With PAD	
		Expression Fold Change	*P* Value	Expression Fold Change	*P* Value
*TNF‐α* (vehicle)	M1	1.50±0.57	0.568	0.77±0.19	0.069
*TNF‐α* (lipopolysaccharide)		1.24±0.30	0.777	1.07±0.19	0.742
*MCP‐1* (vehicle)	M1	4.10±1.26	0.207	0.51±0.10	0.008*
*MCP‐1* (lipopolysaccharide)		1.36±0.34	0.445	0.45±0.09	0.003*
*iNOS* (vehicle)	M1	1.18±0.32	0.663	0.68±0.09	0.023*
*iNOS* (lipopolysaccharide)		1.25±0.25	0.870	1.04±0.24	0.930
*CXCL10* (vehicle)	M1	26.6±12.8	0.273	0.25±0.06	0.043*
*CXCL10* (lipopolysaccharide)		63.9±43.1	0.087	0.50±0.24	0.428
*IL‐10* (vehicle)	M2	2.41±0.75	0.066	1.12±0.27	0.372
*IL‐10* (lipopolysaccharide)		2.11±0.23	0.109	0.39±0.06	0.001*
*CCL17* (vehicle)	M2	1.01±0.35	0.265	1.83±0.52	0.312
*CCL17* (lipopolysaccharide)		0.71±0.26	0.209	1.49±0.51	0.602
*MRC1* (vehicle)	M2	1.67±0.25	0.166	2.49±1.10	0.040*
*MRC1* (lipopolysaccharide)		0.70±0.14	0.124	2.06±0.25	0.016*

Expression fold change was calculated as 2^(−ΔΔCT)^, and represents the fold change in the target gene, normalized to the housekeeping gene and relative to the expression at the baseline time point, reported as mean±SEM. Monocyte‐derived macrophages (MDMs) were stimulated with lipopolysaccharide, to mimic the occurrence of an acute inflammatory event, or vehicle for 24 hours before assessment of gene expression. CCL17 indicates chemokine C‐C motif ligand 17; CXCL10, C‐X‐C motif chemokine 10; IL‐10, interleukin 10; iNOS, inducible nitric oxide synthase; M1, type 1 macrophage; M2, type 2 macrophage; MCP‐1, monocyte chemoattractant protein‐1; MRC1, mannose receptor C type 1; PAD, peripheral artery disease; and TNF‐α, tumor necrosis factor‐α.

* *P*<0.05 by paired Student *t* test within group; denotes a significant change at study end (visit 6) compared with the baseline time point (visit 1).

### Targeted Lipid Mediator Profiling

Plasma lipid mediator analysis was performed in every patient at every time point. Prior work has demonstrated that oral supplementation with n‐3 PUFA increases plasma levels of EPA, DHA, n‐3 DPA, and downstream bioactive lipid mediators, SPMs.^33^ However, it is unknown whether there are baseline differences in the lipid mediator profile of healthy individuals and patients with PAD, and whether short‐term supplementation with an enriched marine oil supplement increases plasma levels of bioactive SPMs or their precursors in a dose‐dependent manner.

By liquid chromatography‐tandem mass spectrometry, plasma levels of 4 essential fatty acids and a total of 58 downstream bioactive lipid mediators were measured. Table 4 details changes in n‐3 and omega‐6 PUFA, the summed values of bioactive lipid mediators grouped into families, total plasma SPM, total SPM:prostaglandin ratio, and n‐3 PUFA:AA ratio (for full biochemical data, including statistical analysis of each individual bioactive mediator, see Tables S5 through S13). At study baseline, a significant difference in PCTR (healthy 1.12±0.47 versus PAD 29.6±13.5, *P*=0.039), n‐3 DPA‐derived RvTs (healthy 2.73±0.87 versus PAD 0.65±0.22, *P*=0.041) and RvDs (healthy 40.7±9.67 versus PAD 6.96±1.69, *P*=0.005), cysteinyl leukotrienes (healthy 1.73±0.67 versus PAD 7.16±2.54, *P*=0.044), SPM:prostaglandin ratio (healthy 115.8±12.4 versus PAD 61.5±10.3, *P*=0.004), and SPM:leukotriene B4 (LTB4) ratio (healthy 11.1±2.87 versus PAD 3.8±1.2, *P*=0.048) was observed between patients with PAD and healthy volunteers.

**Table 4:** Plasma Levels of n‐3 and n‐6 PUFA and Bioactive Lipid Mediator Families Before and After Supplementation

Lipid Mediator Intermediate	Healthy Participants			Patients With PAD			Between‐Group *P* Value (V1)
	Study Start	Study End	*P* Value (Within Group)	Study Start	Study End	*P* Value (Within Group)	
DHA family, pg/mL							
DHA	14 152±2678	30 523±6267	0.006*	15 229±4164	41 418±6251	0.004*	0.827
RvD	23.47±8.09	11.50±3.91	0.251	8.41±3.62	15.87±9.49	0.492	0.120
Protectins	2.20±0.96	2.94±0.76	0.509	1.27±0.20	3.03±0.92	0.088	0.379
PCTR	1.12±0.47	0.84±0.61	0.584	29.61±13.50	10.00±4.53	0.092	0.039*
Maresins	25.04±14.48	19.23±5.85	0.678	15.11±5.34	32.55±7.52	0.139	0.546
MCTR	8.07±3.68	9.99±6.23	0.738	14.20±7.19	21.81±8.56	0.426	0.444
EPA family, pg/mL							
EPA	2861±602	7272±1307	0.002*	2888±1136	11 422±2294	0.011*	0.983
RvE	8.62±3.07	5.66±1.17	0.361	10.39±5.39	15.85±7.76	0.109	0.773
n‐3 DPA family, pg/mL							
n‐3 DPA	2866±643	4929±1012	0.015*	4575±1096	10 260±2602	0.060	0.186
RvT	2.73±0.87	2.66±1.17	0.962	0.65±0.22	1.62±0.69	0.268	0.041*
RvD	40.72±9.67	49.16±12.77	0.604	6.96±1.69	11.11±3.37	0.250	0.005*
Protectins	0.92±0.26	0.21±0.09	0.013*	0.37±0.18	0.60±0.17	0.364	0.112
Maresins	7.61±1.67	54.09±14.17	0.006*	9.93±1.80	136.87±37.44	0.010*	0.357
AA family, pg/mL							
AA	17 300±3981	17 790±3981	0.860	23 267±6370	29 599±5437	0.080	0.389
Lipoxins	31.10±8.32	41.30±9.03	0.427	51.63±14.48	100.89±43.18	0.191	0.224
Leukotrienes	1.64±0.48	4.73±0.98	0.015*	1.34±0.20	6.48±1.29	0.004*	0.590
CysLT	1.73±0.67	0.90±0.48	0.246	7.16±2.54	26.74±19.60	0.372	0.045*
Prostaglandins	1.51±0.29	3.00±0.91	0.139	5.03±2.51	7.71±5.54	0.673	0.159
Thromboxanes	2.09±1.69	0.93±0.63	0.544	7.78±7.68	18.03±17.96	0.434	0.325
Total SPM and ratios							
Total SPM, pg/mL	151.16±16.09	197.59±30.22	0.140	148.53±22.47	350.19±53.81	0.001*	0.911
SPM: Prostaglandin	115.78±12.35	149.70±73.28	0.617	61.45±10.30	415.40±190.05	0.101	0.004*
n‐3:AA	1.14±0.08	2.73±0.28	<0.001*	0.97±0.13	2.19±0.27	0.004*	0.273

Values are expressed as mean±SEM in pg/mL plasma. AA indicates arachidonic acid; CysLT, cysteinyl leukotriene; DHA, docosahexanoic acid; DPA, docosapentaenoic acid; EPA, eicosapentaenoic acid; MCTR, maresin conjugate in tissue regeneration; n‐3, omega‐3; n‐6, omega‐6; PCTR, protectin conjugate in tissue regeneration; PUFA, polyunsaturated fatty acid; RvD, D‐series resolvin; RvE, E‐series resolvin; RvT, 13‐series resolvin; SPM, specialized pro‐resolving lipid mediator; and V, visit number.

* *P*<0.05 by paired or unpaired Student *t* test as appropriate for within‐group or between‐group (healthy vs peripheral artery disease [PAD]) comparisons.

From study start to study end there were significant or nearly significant increases in n‐3 PUFA levels (DHA, EPA, and n‐3 DPA) in both the healthy and PAD groups, but no significant change in AA levels, leading to a significant increase in the n‐3:AA ratio in all patients. There was no observed carryover effect between doses, as the levels of the n‐3 PUFA and AA returned to baseline (statistically equivalent to V1) at the subsequent presupplement visits (V3 and V5). Several individual lipid mediator families significantly increased in either the healthy participants or patients with PAD over the course of the study, most notably the n‐3 DPA‐derived maresins (PAD V1 9.93±1.8 to V6 136.9±37.4, *P*=0.010) and leukotrienes (PAD V1 1.34±0.2 to V6 6.48±1.29, *P*=0.004). There was a significant increase in the total plasma SPM level within the PAD cohort (V1 148.5±22.5 to V6 350.2±53.8, *P*=0.001; Bonferroni adjustment, 0.012). Total prostaglandin and thromboxane levels were unchanged. The plasma total SPM:prostaglandin ratio increased throughout the study and was most notably increased at study termination (V6) in the patients with PAD (V1 61.5±10.3 to V6 415.4±190.1, *P*=0.101). The SPM:LTB4 ratio also increased in the PAD cohort over the course of the study (V1 3.8±1.2 to V6 16.3±6.6, *P*=0.100), while it remained relatively unchanged in the healthy participants (V1 11.1±2.87 to V6 11.44±4.85, *P*=0.943) (Table S13).

Bioactive lipid mediators and SPMs are derived from PUFA via sequential enzymatic actions by lipoxygenase and cyclo‐oxygenase enzymes. These enzymes produce intermediates that may be rate‐limiting for local tissue conversion to the biologically active forms. Thus, we investigated changes in the plasma levels of several key monohydroxylated intermediates of the n‐3 and omega‐6 PUFA following supplementation (Table 5). Notably, in both healthy participants and patients with PAD, all monohydroxylated species significantly increased in a dose‐dependent fashion with the exception of 15‐HETE (no increase), 14‐hydroxy DHA (increase not dose‐dependent), and 7‐hydroxydocosapentaenoic acid (increase not dose‐dependent). Of particular relevance are the notable increases in the precursors 17‐HDHA, which gives rise to RvDs and protectins, 14‐hydroxy DHA (which is the maresin family pathway marker), and 18‐hydroxy EPA (which gives rise to the RvEs).

**Table 5:** Plasma Levels of Monohydroxylated Lipid Mediator Pathway Markers Before and After Supplementation

Lipid Mediator Intermediate	Healthy Participants			Patients With PAD			Between‐Group *P* Value (V1)
	Study Start	Study End	*P* Value (Within Group)	Study Start	Study End	*P* Value (Within Group)	
DHA family, pg/mL							
17‐HDHA	17.26±3.59	161.66±36.62	0.002*	40.25±5.85	394.77±79.36	0.002*	0.003*
14‐HDHA	7.52±0.83	81.69±16.48	0.001*	13.12±1.94	150.12±27.40	0.002*	0.013*
7‐HDHA	8.01±1.56	50.06±8.33	<0.001*	10.28±1.21	89.61±20.72	0.007*	0.273
4‐HDHA	8.75±1.42	67.15±9.05	<0.001*	14.52±4.46	141.34±31.57	0.005*	0.215
EPA family, pg/mL							
18‐HEPE	24.03±4.34	665.46±151.31	0.002*	51.58±17.78	1130.16±346.52	0.015*	0.132
15‐HEPE	15.32±3.26	105.88±22.79	0.002*	45.97±10.94	249.50±56.96	0.006*	0.012*
5‐HEPE	34.94±9.40	223.99±44.86	0.003*	46.03±17.15	405.38±104.10	0.010*	0.567
n‐3 DPA family, pg/mL							
17‐HDPA	35.66±2.99	252.29±43.43	0.006*	62.05±11.40	400.73±72.79	0.002*	0.031*
14‐HDPA	6.77±0.71	154.90±34.45	0.002*	10.41±1.82	208.55±41.09	0.003*	0.069
13‐HDPA	2.17±0.70	25.80±1.97	<0.001*	3.20±0.80	41.66±10.80	0.013*	0.250
7‐HDPA	7.14±1.61	12.43±1.97	0.051	4.07±1.24	19.91±4.09	0.002*	0.156
AA family, pg/mL							
15‐HETE	100.71±19.16	101.05±21.39	0.979	256.20±66.08	261.94±51.14	0.607	0.030*
5‐HETE	39.37±9.30	82.84±9.15	0.021*	37.30±5.61	112.22±25.41	0.022*	0.855

Values are expressed as mean±SEM in pg/mL plasma. AA indicates arachidonic acid; DHA, docosahexanoic acid; DPA, docosapentaenoic acid; EPA, eicosapentaenoic acid; HDHA, hydroxydocosahexaenoic acid; HDPA, hydroxydocosapentaenoic acid; HEPE, hydroxyeicosapentaenoic acid; HETE, hydroxyeicosatetraenoic acid; PAD, peripheral artery disease; and V, visit number.

* *P*<0.05 by paired or unpaired Student *t* test as appropriate for within‐group or between‐group comparisons.

**Figure 2:**
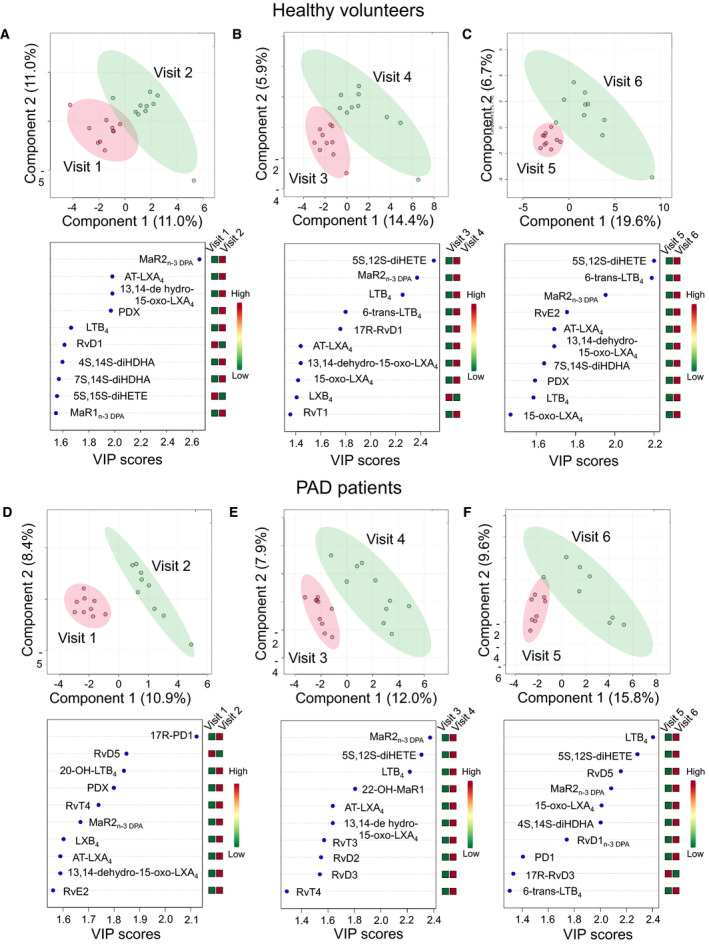
**Lipid mediator (LM) profiles were investigated in (A through C) healthy volunteers and (D through F) patients with peripheral artery disease (PAD) presupplementation (visit [V] 1, V3, and V5) and postsupplementation (V2, V4, and V6). Results were interrogated using partial least squares discriminant analysis following auto‐scaling (mean‐centered and divided by the SD of each variable).** The colored area represents the 95% CI. (*Top panels*) Score plots and (*bottom panels*) plots displaying the LM with the 10 highest variable importance in projection (VIP) scores from component 1. Results are representative of n=10 for healthy volunteers and, n=8 to 10 for patients with PAD V1 to V2, n=10 for patients with PAD V3 to V6.

To gain greater insight into the differences in the lipid mediator profiles of healthy participants and patients with PAD and how this is affected by oral enriched marine oil supplementation, a PLS‐DA of the lipid mediator concentrations before and after each dosing period were conducted, as shown in Figure 2. The PLS‐DA model effectively reduces the dimensionality of the data set, allowing observations regarding which lipid species contribute most to the variance between the healthy and PAD groups before and after supplementation, as shown in the variable importance in projection scores. In addition, the score plots allow for determination regarding whether there is a significant difference in the overall lipid mediator profile of the healthy and PAD groups at each visit. These results demonstrate that lipid mediator pathways upregulated in patients with PAD are distinct from those observed from healthy volunteers. Morever, short‐term supplementation with the lipid emulsion produced a significant shift (V1 versus V2) in the lipid mediator profiles in both groups. This pattern is seen in both groups between subsequent visits, demonstrating a consistent shift in the lipid mediator profiles over each dosing interval, ie, from V3 to V4 and V5 to V6, respectively.

### Dose‐Dependent Analysis

To address the question of which dose of the supplement led to the greatest biochemical changes, a linear mixed model of the SPM:prostaglandin ratio was investigated. When compared with the baseline, presupplementation, value, there was a significant difference in this ratio for all doses and in both the healthy and PAD subgroups individually. When comparing the doses with one another, the 60 mL dose led to a significantly greater increase in the ratio of SPM:prostaglandin than either the 15 or 30 mL doses (60 mL versus 15 mL estimate: 0.64±0.18, *P*=0.001; 60 mL versus 30 mL estimate: 0.50±0.18, *P*=0.005).

**Figure 3:**
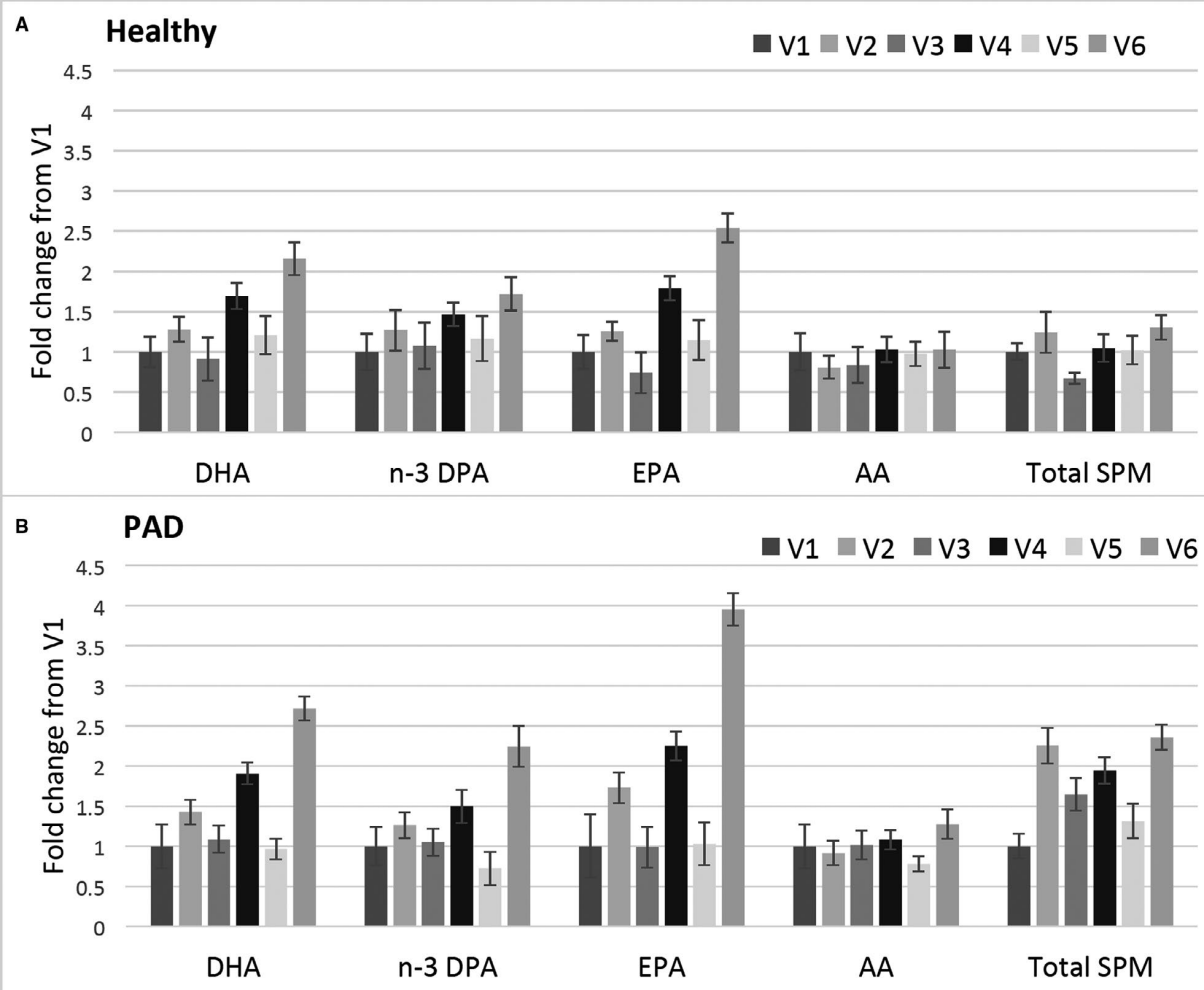
**Dose‐dependent changes for omega‐3 (n‐3) fatty acids (docosahexanoic acid [DHA], n‐3 docosapentaenoic acid [DPA], eicosapentaenoic acid [EPA]), arachidonic acid (AA), and total specialized pro‐resolving lipid mediators (SPMs) for healthy participants (A) and patients with peripheral artery disease (B).** V indicates visit number.

Similar analyses were conducted for dose‐dependent effects on the monohydroxylated metabolites. The following precursor molecules demonstrated a significant dose‐dependent increase with supplementation, with the 60 mL dose leading to the most significant increase: 17‐HDHA, 17‐hydroxydocosapentaenoic acid, 13‐hydroxydocosapentaenoic acid, and 18‐hydroxy EPA (not shown). The dose‐dependent changes of the n‐3 PUFAs, AA, and total SPM are illustrated in Figure 3.

### Correlation Between Plasma Lipid Mediators and Leukocyte Phenotype

To assess the association between leukocyte outcomes (phagocytic activity and cell surface marker expression) and biochemical predictors (total SPM and total SPM:prostaglandin ratio) at each visit, a linear mixed model with random intercepts was generated (Table 6). Within both the healthy participants and patients with PAD, as total plasma SPMs increased, there were statistically significant–associated decreases in the monocyte surface markers CD18, CD163, and CD36. Within the PAD population, as total plasma SPMs increased, both CD54 and chemokine receptor 2 expression decreased. Within the healthy individuals, as the SPM:prostaglandin ratio increased, there were associated decreases in the monocyte surface markers CD18 and CD36 and an increase in neutrophil phagocytosis. Within the PAD population, as the SPM:prostaglandin ratio increased, there were associated decreases in the monocyte markers CD18, CD36, CD163, CD54, and chemokine receptor 2 (Table 6).

**Table 6:** Total Plasma SPM and SPM:Prostaglandin Ratio as Predictor of Change in Leukocyte Phenotype

Leukocyte Phenotype	Plasma SPM			Plasma SPM:Prostaglandin Ratio		
	Estimate of Change*	95% CI	*P* Value	Estimate of Change*	95% CI	*P* Value
Healthy participants						
Monocyte CD18	−7.1	−11.5 to −2.73	0.002^†^	−10.2	−15.1 to −5.35	<0.001^†^
Monocyte CD163	−2.39	−4.03 to −0.75	0.005^†^	−2.20	−4.49 to 0.09	0.060
Monocyte CD36	−10.4	−18.1 to −2.77	0.009^†^	−13.2	−22.6 to −3.74	0.007^†^
Neutrophil phagocytosis	0.04	−0.003 to 0.09	0.066	0.064	0.007–0.12	0.028^†^
Monocyte CD49d	0.60	−0.73 to 1.92	0.372	0.068	−1.57 to 1.68	0.944
Monocyte CCR2	0.33	−2.78 to 3.45	0.830	1.29	−2.77 to 5.35	0.525
Monocyte phagocytosis	0.003	−0.03 to 0.03	0.848	0.024	−0.02 to 0.06	0.258
Monocyte CD54	0.06	−1.39 to 1.51	0.934	−0.53	−2.19 to 1.14	0.528
Patients with PAD						
Monocyte CD18	−10.3	−13.7 to −6.8	<0.001^†^	−19.1	−27.6 to −10.6	<0.001^†^
Monocyte CD36	−26.1	−36.8 to −15.4	<0.001^†^	−73.3	−94.7 to −51.9	<0.001^†^
Monocyte CD163	−3.96	−5.68 to −2.24	<0.001^†^	−6.01	−10.0 to −1.98	0.004^†^
Monocyte CD54	−2.63	−4.14 to −1.13	0.001^†^	−6.07	−9.39 to −2.75	<0.001^†^
Monocyte CCR2	−4.0	−7.62 to −0.37	0.032^†^	−8.19	−16.1 to −0.30	0.042^†^
Monocyte CD49d	−0.41	−1.54 to 0.72	0.472	−0.40	−2.05 to 2.86	0.743
Neutrophil phagocytosis	0.002	−0.05 to 0.05	0.928	0.06	−0.06 to 0.18	0.315
Monocyte phagocytosis	<0.001	−0.03 to 0.03	0.965	0.009	−0.06 to 0.08	0.793

CCR2 indicates chemokine receptor 2; CD, cluster of differentiation; and PAD, peripheral artery disease.

* For every unit increase in total specialized pro‐resolving lipid mediator (SPM) or SPM:prostaglandin ratio, there is an associated change in the leukocyte phenotype by the given estimate.

^†^ *P*<0.05. The leukocyte phenotypes are ordered from most statistically significant to least, based on unit change in total plasma SPM.

## Discussion

**Figure 4:**
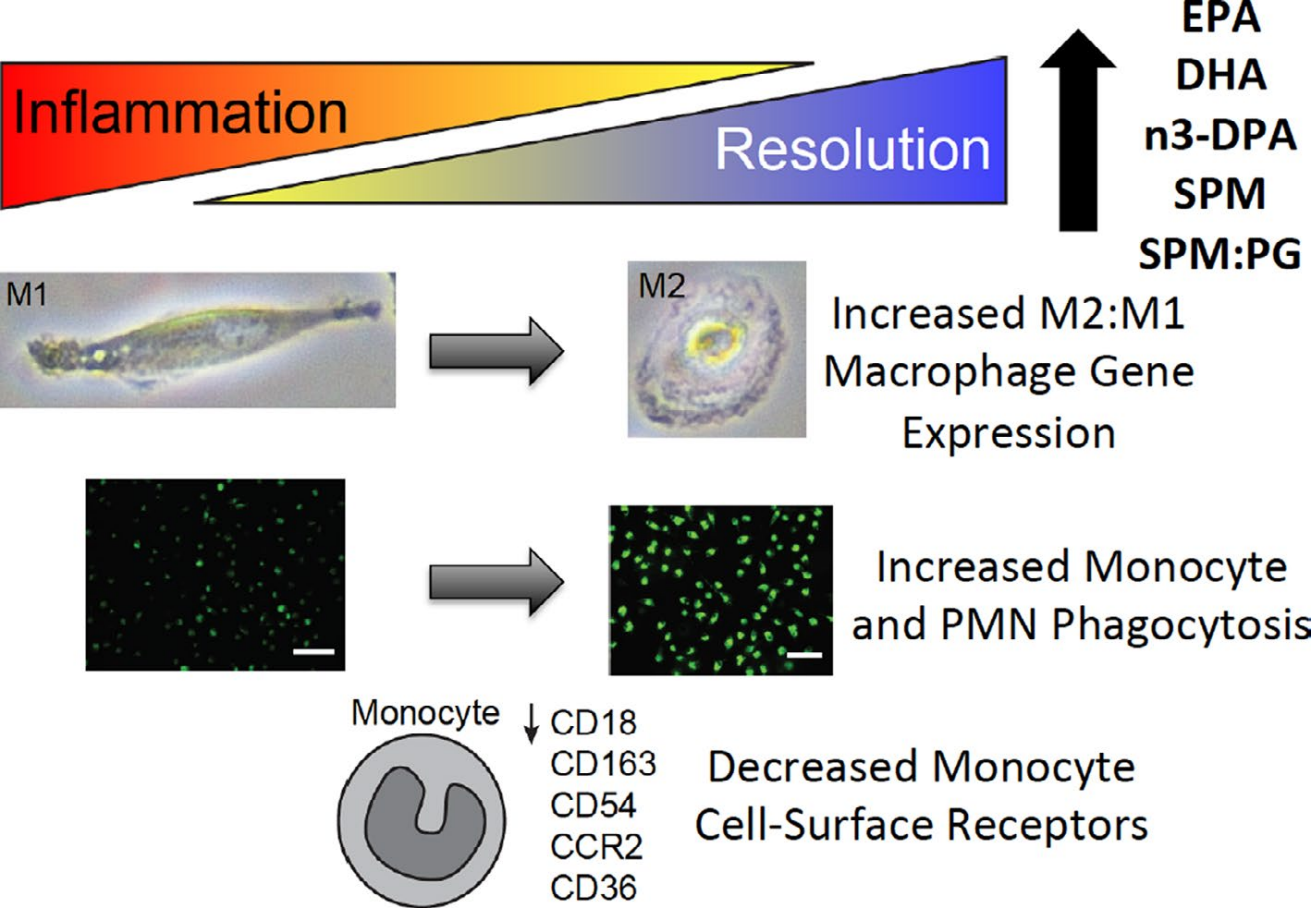
**Illustration of the changes in lipid mediator biochemistry and leukocyte (monocyte and monocyte‐derived macrophage) phenotype in patients with peripheral artery disease with marine oil supplementation**. **The balance shifts from inflammation towards resolution**. CCR2 indicates chemokine receptor 2; CD, cluster of differentiation; DHA, docosahexanoic acid; DPA, docosapentaenoic acid; EPA, eicosapentaenoic acid; M1, type 1 macrophage; M2, type 2 macrophage; n‐3, omega‐3; and SPM, specialized pro‐resolving lipid mediators.

This is the first study of its kind to investigate the metabolic, biochemical, and cellular impact of a short course of an enriched marine oil supplement on healthy individuals and patients with PAD. We hypothesize that PAD is associated with an altered balance of inflammation‐resolution that may be targeted by lifestyle and nutritional interventions. At entry, patients with PAD demonstrated higher levels of inflammatory cytokines, greater type 1 macrophage versus type 2 macrophage–related MDM gene expression, reduced n‐3 index, and reduced plasma SPM:prostaglandin and SPM:LTB4 ratios compared with healthy individuals. Over the course of treatment, leukocyte profiling demonstrated a shift towards a less inflammatory and more pro‐resolving phenotype, most notably within the PAD cohort. Supplementation led to an increase in phagocytic activity of peripheral blood monocytes and neutrophils, a process that plays an important role in the shift from inflammation to resolution.^34^ Peripheral blood monocytes demonstrated an altered cell surface marker expression, away from those associated with inflammation and atherosclerotic disease. Gene expression patterns in MDM from patients with PAD displayed a less inflammatory (type 1 macrophage) and greater reparative (type 2 macrophage) phenotype after supplementation, demonstrating notable transcriptional remodeling after the short treatment course. These cellular alterations are illustrated in Figure 4. The PLS‐DA demonstrates that lipid mediator pathways upregulated in patients with PAD are distinct from those observed in healthy volunteers, suggesting that lipid mediator pathways are differentially regulated postsupplementation in these 2 groups. Given that statistically significant increases in select lipid mediators were observed, and that these concentrations are within the bioactive ranges of these molecules,^14^, ^35^, ^36^, ^37^, ^38^ this suggests that upregulation in these molecules may be functionally relevant in the observed protective actions on leukocytes. Finally, these data demonstrate strong associations between integrated biochemical measures of lipid mediators (eg, plasma SPM:prostaglandin ratio) and phenotypic changes in circulating leukocytes in both healthy participants and patients with PAD. Taken together these data suggest that short‐term enriched marine oil supplementation dramatically remodels downstream lipid mediator pathways and induces a less inflammatory and more pro‐resolution phenotype in circulating leukocytes and MDM.

Recent work has demonstrated the important role for n‐3 PUFA and SPMs in vascular health and inflammation.^39^, ^40^ Unresolved inflammation plays a central role in the progression of atherosclerosis and symptomatic vascular disease.^41^ Unstable atherosclerotic plaques, which are more likely to lead to acute vascular disease such as heart attacks and strokes, have been found to contain an imbalance between proinflammatory mediators, such as inflammatory leukotrienes, and pro‐resolution mediators, such as resolvins, compared with more stable plaques.^18^ Maresins have been shown to improve the hemostatic function of platelets while suppressing their inflammatory function.^42^ SPMs have an atheroprotective action during vascular injury and can reduce neointimal hyperplasia and leukocyte trafficking to injured arteries.^43^, ^44^ These findings suggest that unchecked, chronic inflammation such as that seen in vascular diseases including PAD may be related to reduced bioavailability of the mediators that drive resolution.

Current clinical practice guidelines include the use of antiplatelet agents and statins in patients with PAD, as reflected in our pilot study population. It is important to note that both aspirin and statins have been demonstrated to impact SPM biosynthetic pathways. Specifically, acetylation of COX‐2 by aspirin promotes the generation of epimeric lipoxins and resolvins (“aspirin‐triggered” forms), which may have a longer biologic half‐life than the native mediators.^45^, ^46^ Statins increase S‐nitrosylation of COX‐2, which promotes epimeric lipoxin production as well as the generation of RvTs derived from n‐3 DPA.^47^, ^48^ Thus, the use of these agents would be expected to alter the biochemical and cellular responses to n‐3 PUFA supplementation, and to favor resolution.^49^, ^50^ Large‐scale studies of n‐3 supplementation in patients with cardiovascular disease have yielded mixed results with a variety of inclusion criteria, formulations, and dosing employed. The recent REDUCE‐IT (Reduction of Cardiovascular Events with Icosapent Ethyl–Intervention Trial) tested a specific EPA formulation (icosapent ethyl, 4 g/d) in patients with established cardiovascular disease or diabetes mellitus and with elevated triglyicerides (>135 mg/dL) already taking statin therapy.^21^ The study demonstrated a 25% to 31% reduction in first and all major cardiovascular events over a median follow‐up of 5 years. It remains unclear how these prior studies relate specifically to PAD, and what the optimal formulation of marine oil supplementation would be to augment SPM pathways either short‐ or long‐term in specific populations.

Little is known regarding differences in the lipid mediator profiles of healthy individuals and those with PAD. There was no difference in plasma levels of EPA or DHA at baseline, although the patients with PAD had a significantly lower n‐3 index. This indicates that peripheral blood cellular membranes in patients with PAD were composed of a greater proportion of other fatty acids, such as saturated or omega‐6 fatty acids. At baseline, patients with PAD had significantly higher levels of some monohydroxylated metabolites, such as 17‐HDHA and 14‐hydroxy DHA , while having lower levels of several bioactive SPMs such as the RvDs and n‐3 DPA‐derived RvTs and RvDs. This could potentially signify baseline differences in dietary intake, an enzymatic defect, or an alternatively preferred pathway in the biochemical route to SPM production. It has been shown in murine models that the activity of 15‐lipoxygenase, one of the key enzymes involved in SPM production, can promote either atherogenesis or SPM synthesis dependent on the proportion of different fatty acids in the diet.^51^, ^52^ Leukocytes from obese patients have been found to lack efficient conversion of DHA to SPM, and this can be corrected in the resolvin pathway by the addition of 17‐HDHA.^53^

With supplementation, there was a significant and dose‐dependent increase in monohydroxylated PUFA metabolites throughout both healthy participants and patients with PAD, possibly as a result of the supplement itself containing these biochemical intermediates. Lipid mediators, as classic autocoids, are thought to be largely generated locally at sites of tissue injury and rapidly degraded. Notably, recent studies have demonstrated that isolated vascular cells and tissues can convert requisite precursors such as DHA and 17‐HDHA into bioactive SPMs.^54^ There was also a significant increase in the total plasma SPMs and in the SPM:prostaglandin ratio, particularly within the PAD cohort. These findings suggest that short‐term dietary intake of enriched marine oil can dramatically alter the plasma profile of bioactive lipid mediators in PAD.

This pilot study helps to establish a framework for further research into the impact of treatment with concentrated n‐3 PUFA and their metabolites targeting resolution pathways in humans. It has been shown in animal models that systemic delivery of SPMs can delay progression of atherosclerosis and prevent progression to vulnerable plaque types.^18^, ^55^, ^56^ Prior work from our group has demonstrated that local vascular delivery of SPMs can lead to a decrease in neointimal hyperplasia after vascular injury.^43^, ^44^, ^57^ Questions remain regarding the precise biological role for many of these SPMs in humans, and the mechanisms by which they may be dysregulated in atherosclerotic disease, either in synthesis or via receptor‐mediated actions, are under investigation. Key limitations of this pilot study include its modest sample size, unblinded and short‐term design, and lack of clinically meaningful end points. The results presented in this pilot study are not controlled for potential confounders because of small sample size constraints. The *P* values generated in this pilot study were not adjusted for multiple testing that protect against false positives, and thus the results in this study need to be interpreted with caution until they can be confirmed in a larger study. Additionally, there may exist a modest carryover effect in our study design, as was seen with the n‐3 index, between the doses. Further clinical studies will be enhanced by the use of surrogate biomarkers as demonstrated here, complemented by target organ imaging to assess changes in disease state or plaque morphology.

## Conclusions

This study provides a foundation for characterizing biochemical and cellular biomarkers of inflammation and resolution in PAD, with future work aiming to correlate upstream (eg, nutritional or pharmacologic) interventions, immune cell function, and downstream clinical events.

## Sources of Funding

This study was supported by a research grant from Metagenics Inc. and the National Institutes of Health (HL119508) to Conte. This work was also supported by funding from a Sir Henry Dale Fellowship jointly funded by the Wellcome Trust and the Royal Society (grant 107613/Z/15/Z), funding from the European Research Council under the European Union's Horizon 2020 Research and Innovation Programme (grant number: 677542) and the Barts Charity (grant number: MGU0343) to Dalli. The sponsor played no part in the implementation or analysis of the study, authorship of the article, or decision to publish the results.

## Disclosures

Conte is co‐inventor on patents (US Patent and Trademark Office numbers 9,463,177 and 10,111,847) assigned to Regents of the University of California and Brigham and Women's Hospital and co‐founder of VasaRx, and reports research support from Metagenics Inc. The remaining authors have no disclosures to report.

## Supporting information


**Tables S1–S13**
**Figures S1–S2**
