# Supplementary material for: Treatment With a Marine Oil Supplement Alters Lipid Mediators and Leukocyte Phenotype in Healthy Patients and Those With Peripheral Artery Disease
Source: J Am Heart Assoc. 2020 Jul 22;9(15):e016113. doi: 10.1161/JAHA.120.016113 (PMC7792251; doi:10.1161/JAHA.120.016113)
Supplement: Supplementary file 1 — Tables S1–S13 Figures S1–S2 [file JAH3-9-e016113-s001.pdf]

SUPPLEMENTAL MATERIAL

Table S1. Inclusion and Exclusion criteria

**Inclusion criteria**	**Exclusion criteria**
PAD Patients:<br>1. Mild to severe claudication (Rutherford 1-3)<br>2. Resting or exercise ABI <0.9 or TBI <0.6<br>3. Age 40 and more<br>Healthy Volunteers:<br>1. Age 20-80	PAD Patients and healthy volunteers:<br>1. Plan to undergo surgical procedure or PVI for treatment of PAD within one month<br>2. Evidence of active infection<br>3. Hypersensitivity or allergy to fish or seafood<br>4. Already on n-3 PUFA or equivalent<br>5. Chronic liver disease, end-stage renal disease, or chronic inflammatory disorders<br>6. Poorly controlled diabetes (HbA1c > 8%)<br>7. Recent other major surgery or illness within 30 days<br>8. Use of immunosuppressive medications or steroids<br>9. History of organ transplantation<br>10. Pregnancy, or plans to become pregnant, or lactating<br>Healthy Volunteers:<br>1. hsCRP > 2mg/L<br>2. Regular aspirin use<br>3. Regular non-steroidal anti-inflammatory drug use

PAD, peripheral arterial disease; ABI, ankle brachial index; TBI, toe brachial index, PVI, peripheral vascular intervention; PUFA, polyunsaturated fatty acids; BMI, body mass index; hsCRP, high-sensitivity C-reactive protein

Table S2. Essential fatty acid and SPM precursor concentrations (free fatty acid form) in the 15mL supplement.

	Supplement		
**DHA Bioactive Metabolome**	Mean	±	SEM
17-HDHA	1.01	±	0.07
14-HDHA	0.78	±	0.04
7-HDHA	0.55	±	0.03
4-HDHA	0.25	±	0.01
DHA	13.26	±	1.60
**n-3 DPA Bioactive Metabolome**			
17-HDPA	0.74	±	0.04
14-HDPA	0.20	±	0.01
13-HDPA	0.08	±	0.01
7-HDPA	0.01	±	0.00
DPA	5.73	±	0.39
**EPA Bioactive Metabolome**			
18-HEPE	1.70	±	0.15
15-HEPE	0.61	±	0.06
12-HEPE	0.50	±	0.05
5-HEPE	6.54	±	0.48
EPA	11.85	±	2.91
**AA Bioactive Metabolome**			
15-HETE	0.05	±	0.01
12-HETE	0.04	±	0.00
5-HETE	0.80	±	0.08
AA	0.79	±	0.10

The free fatty acid concentrations of SPM precursors and substrates were determined in the marine oil supplement. Results are expressed as μg/15 ml which, for the marine oil supplement, is equivalent to the 1.5-gram dose. Results are mean ± SEM of 4 determinations.

Table S3. Change in monocyte and neutrophil phagocytosis of E. Coli over the study period.

	**Healthy Subjects**			**PAD Subjects**		
Phagocyte	Study Start	Study End	P Value	Study Start	Study End	P Value
Monocytes	55 ±1.3	58 ±2.9	0.357	58 ±3.7	64 ±2.9	0.014*
Neutrophils	63 ±2.7	75 ±3.8	0.002*	66 ±5.2	73 ±5.8	0.176

Values are given in normalized median fluorescence intensity (MFI), reported as mean ±SEM. *P<0.05 by paired Student's t test (within group).

Table S4. Differences in monocyte-derived macrophage (MDM) gene expression in PAD versus healthy subjects at baseline

		**PAD Subjects Relative to Healthy Subjects**	
Gene	M1 or M2	Expression Fold Change	P Value
TNFα (veh)	M1	2.71	0.02*
TNFα (LPS)		0.81	0.33
MCP-1 (veh)	M1	1.92	0.05*
MCP-1 (LPS)		1.91	0.01*
iNOS (veh)	M1	1.34	0.26
iNOS (LPS)		1.04	0.94
CXCL10 (veh)	M1	7.87	0.04*
CXCL10 (LPS)		1.81	0.29
IL-10 (veh)	M2	1.50	0.19
IL-10 (LPS)		2.72	0.009*
CCL17 (veh)	M2	0.48	0.09
CCL17 (LPS)		0.22	0.14
MRC1 (veh)	M2	0.52	0.09
MRC1 (LPS)		0.30	0.01*

Expression fold change was calculated as $2^{(-\Delta\Delta CT)}$, and represents the fold change in the average expression of the target gene within the PAD cohort versus the average expression of the target gene within the healthy subject cohort, normalized to the housekeeping gene (HPRT), at the baseline time point. MDM were stimulated with lipopolysaccharide (LPS), to mimic the occurrence of an acute inflammatory event, or vehicle (veh) for 24 hours prior to assessment of gene expression. *$P<0.05$ by unpaired Student's t test. TNFα denotes tumor necrosis factor alpha; MCP-1, monocyte chemoattractant protein-1; iNOS, inducible nitric oxide synthase; CXCL10, C-X-C motif chemokine 10; IL-10, interleukin 10; CCL17, chemokine (C-C motif) ligand 17; MRC1, mannose receptor C Type 1; M1, type 1 macrophage (inflammatory); M2, type 2 macrophage (pro-resolution).

Table S5. Plasma levels of DHA metabolome lipid mediators by study visit, healthy subjects.

All values pg/ml plasma Avg±SEM	Healthy					
DHA Metabolome	V1	V2	V3	V4	V5	V6
**n-3 Fatty Acid**						
DHA	14152.12±2678.45	18101.14±2855.83	12883.88±3443.06	23951.48±3870.11**	17105.55±4076.01	30523.66±6267.29***~
**Precursors**						
17-HDHA	17.26±3.59	67.30±14.03*	18.41±4.23	80.42±19.22**	19.95±3.46	161.66±36.62***~
14-HDHA	7.52±0.83	34.05±6.56*	9.75±1.61	37.41±6.87**	11.52±2.79	81.69±16.48***~
7-HDHA	8.01±1.56	19.84±2.64*	8.70±2.44	27.09±5.27**	8.71±1.44	50.06±8.33***~
4-HDHA	8.75±1.42	28.63±4.27*	9.76±1.85	42.22±6.09**	12.01±2.84	67.15±9.05***~
**Bioactive LMs**						
RvD1	6.39±3.38	0.58±0.35	5.72±3.92	2.14±0.85	1.97±1.09	1.62±0.48
RvD2	0.60±.0.28	0.70±0.40	0.68±0.32	0.63±0.34	1.85±0.74	1.03±0.49***
RvD3	0.27±0.12	0.31±0.18	0.30±0.14	0.28±0.15	0.83±0.33	0.46±0.22***
RvD4	14.01±6.92	4.19±2.62	3.84±1.65	2.89±1.19	3.08±0.71	7.13±3.62
RvD5	0.29±0.13	0.13±0.07	0.34±0.16	0.61±0.20	0.26±0.12	0.14±0.09
RvD6	0.13±0.05	0.46±0.21	0.24±0.09	0.56±0.22	0.03±0.02	0.31±0.14
17R-RvD1	1.63±0.70	1.67±0.84	0.44±0.18	1.24±0.38	3.03±1.90	0.66±0.41~
17R -RvD3	0.15±0.10	0.20±0.13	0.24±0.12	0.33±0.11	0.46±0.16	0.16±0.11
PD1	0.51±0.19	0.61±0.21	0.66±0.22	0.78±0.21	0.68±.17	0.94±0.32
10S,17S-diHDHA	0.07±0.04	0.32±0.11	0.17±0.07	0.30±0.09	0.11±0.04	0.47±0.15***~
17R -PD1	0.13±0.04	0.24±0.07	0.09±0.04	0.11±0.04	0.12±0.03	0.15±0.03
22-OH-PD1	1.50±0.92	0.93±40	1.23±0.63	0.97±0.22	1.63±1.01	1.38±0.57
PCTR1	0.00±0.00	0.00±0.00	3.17±3.17	1.58±1.58	6.25±5.49	0.00±0.00
PCTR2	0.54±0.31	0.33±0.25	0.17±0.17	0.00±0.00	0.10±0.10	0.00±0.00
PCTR3	0.58±0.44	0.25±0.25	0.50±0.33	0.91±0.61	2.28±1.92	0.84±0.61
MaR1	1.85±0.74	2.33±0.99	3.91±1.26	3.43±1.26	2.14±0.87	6.07±2.84
MaR2	1.18±0.41	2.39±1.27	0.62±0.27	1.20±0.51	0.59±0.20	0.87±0.21
22-OH-MaR1	21.45±14.80	41.66±32.35	2.91±1.20	24.82±19.02	28.77±20.74	9.83±6.14
7S,14S-diHDHA	0.47±0.47	6.75±3.75	1.81±0.94	0.69±0.51	0.00±0.00	1.76±0.72***
4S,14S-diHDHA	0.09±0.05	0.36±0.15	0.29±0.16	0.56±0.22	0.10±0.07	0.70±0.39
14-oxo-MaR1	0.00±0.00	0.00±0.00	0.00±0.00	0.00±0.00	0.00±0.00	0.00±0.00
MCTR1	6.79±3.63	1.74±1.74	4.72±3.44	4.53±3.52	17.02±8.61	9.08±6.15
MCTR2	0.59±0.20	0.84±0.50	0.51±0.45	0.46±0.25	0.42±0.22	0.23±0.23
MCTR3	0.69±0.41	0.73±0.53	0.62±0.34	0.79±0.63	1.10±0.44	0.68±0.43
**Mediator Families**						

RvD	23.47±8.09	8.24±2.51	11.80±3.86	8.68±1.79	11.51±2.17	11.50±3.91
Protectins	2.20±0.96	2.10±.047	2.15±0.64	2.16±0.19	2.54±1.05	2.94±0.76
PCTR	1.12±0.47	0.58±0.32	3.85±3.12	2.49±1.60	8.64±5.57	0.84±0.61
Maresins	25.04±14.48	53.49±31.68	9.53±2.62	30.71±18.75	31.60±20.59	19.23±5.85
MCTR	8.07±3.68	3.31±2.12	5.86±3.36	5.79±3.55	18.54±8.54	9.99±6.23

Within group

*p<0.05 vs V1

**p<0.05 vs V3

***p<0.05 vs V5

~p<0.05 V1 vs. V6

Table S6. Plasma levels of DHA metabolome lipid mediators by study visit, PAD subjects.

All values pg/ml plasma Avg±SEM	PAD					
DHA Metabolome	V1	V2	V3	V4	V5	V6
**n-3 Fatty Acid**						
DHA	15229.78±4164.68	21731.86±3329.06*	16553.96±2808.40	29024.83±3996.97**	14671.90±1907.97	41418.66±6251.12***~
**Precursors**						
17-HDHA	40.25±5.85^	129.39±27.08*	52.75±11.16	196.46±45.50**	52.41±9.32	394.77±79.36***~
14-HDHA	13.12±1.94^	335.63±283.64	140.36±124.44	84.61±21.77	19.66±4.22	150.12±27.40***~
7-HDHA	10.28±1.21	33.89±6.41*	14.27±2.63	47.28±10.79**	14.78±2.24	89.61±20.72***~
4-HDHA	14.52±4.46	38.98±6.27*	15.49±2.61	74.74±12.85**	16.09±2.43	141.34±31.57***~
**Bioactive LMs**						
RvD1	1.13±0.43	1.14±0.76	0.66±0.22	0.82±0.24	0.59±0.23	9.70±8.60
RvD2	0.67±0.19	0.58±0.23	0.26±0.12	0.89±0.30	0.70±0.34	0.45±0.19
RvD3	0.30±0.08	0.26±0.11	0.11±0.05	0.40±0.13	0.31±0.15	0.20±0.08
RvD4	4.19±2.38	2.86±1.16	1.25±0.32	1.39±0.34	3.16±1.49	3.07±1.68
RvD5	0.59±0.20	0.05±0.05	0.61±0.22	0.31±0.24	0.20±0.12	0.97±0.17
RvD6	0.36±0.14	0.39±0.14	0.18±0.10	0.15±0.10	0.16±0.07	0.40±0.18***
17R-RvD1	1.02±0.52	1.38±1.10	0.59±.059	1.23±0.55	0.58±0.50	1.03±0.48
17R -RvD3	0.16±0.10	0.12±0.08	0.19±0.11	0.17±0.08	0.21±0.09	0.05±0.05
PD1	0.76±0.22	0.59±0.23	0.51±0.09	1.05±0.33	0.39±0.17	0.78±0.18
10S,17S-diHDHA	0.13±0.08	0.44±0.12*	0.38±0.17	0.96±0.36	0.11±0.08	0.39±0.20
17R -PD1	0.13±0.04	0.36±0.07*	0.17±0.06	0.27±0.05	0.21±0.07	0.27±0.08~
22-OH-PD1	0.25±0.17	0.68±0.27	0.47±0.31	0.99±0.29	0.71±0.55	1.59±0.85
PCTR1	23.97±19.93	21.36±18.54	52.34±26.43	14.36±8.04	18.08±12.34	2.81±2.81
PCTR2	0.00±0.00	0.00±0.00	0.17±0.17	0.16±0.16	0.00±0.00	0.00±0.00
PCTR3	5.64±3.29	2.11±1.15	4.55±1.93	4.33±2.31	5.08±2.50	7.18±2.88
MaR1	5.88±2.88	1.68±.069	5.89±2.69	11.53±6.76	4.09±2.20	8.01±2.85
MaR2	2.20±0.98	1.88±0.79	1.56±0.56	2.25±0.57	2.94±0.77	3.52±1.10
22-OH-MaR1	2.09±1.22	2.63±0.95	1.16±0.93	4.95±1.24**	6.44±5.19	12.99±6.25***
7S,14S-diHDHA	4.72±3.40	5.97±4.49	7.03±4.05	10.52±7.02	12.12±11.28	6.27±2.74
4S,14S-diHDHA	0.31±0.22	0.79±0.35	0.40±0.12	0.66±0.13	0.30±0.16	1.76±0.46***~
14-oxo-MaR1	0.00±0.00	0.00±0.00	0.00±0.00	0.00±0.00	0.00±0.00	0.00±0.00
MCTR1	9.00±6.14	0.00±0.00	7.50±5.85	6.51±3.49	2.23±2.23	6.09±4.46
MCTR2	0.77±0.32	0.77±0.42	1.51±0.65	1.90±0.91	5.61±3.40	5.81±4.45
MCTR3	4.44±2.38	1.88±1.46	4.60±3.41	4.19±2.00	10.11±3.83	9.91±3.65
**Mediator Families**						

RvD	8.41±3.62	6.78±2.30	3.85±0.71	5.36±0.81	5.92±1.39	15.87±9.49
Protectins	1.27±0.20	2.08±0.41	1.53±0.35	3.26±0.46**	1.42±0.60	3.03±0.92
PCTR	29.61±13.50^	23.46±18.30	57.06±27.14	18.85±9.28	23.17±11.88	10.00±4.53
Maresins	15.11±5.34	12.95±4.76	16.05±7.11	29.90±13.45	25.89±12.18	32.55±7.52
MCTR	14.20±7.19	2.66±1.70	13.61±6.73	12.60±3.81	17.95±6.72	21.81±8.56

Within group

*p<0.05 vs V1

**p<0.05 vs V3

***p<0.05 vs V5

~p<0.05 V1 vs. V6

Between Groups

^p<0.05 vs Healthy at V1

Table S7. Plasma levels of n-3 DPA metabolome lipid mediators by study visit, healthy subjects.

All values pg/ml plasma Avg±SEM	Healthy					
n-3 DPA Metabolome	V1	V2	V3	V4	V5	V6
**n-3 Fatty Acid**						
n-3 DPA	2866.77±643.54	3637.70±922.10	3088.60±889.71	4209.86±622.79	3340.45±940.54	4929.74±1012.83***~
**Precursors**						
17-HDPA	35.66±2.99	127.66±21.80*	32.87±6.57	145.34±25.48**	42.08±6.37	252.29±43.43***~
14-HDPA	6.77±0.71	64.72±13.38*	6.64±0.84	79.90±16.42**	8.25±1.46	154.90±34.45***~
13-HDPA	2.17±0.70	11.70±1.96*	2.33±0.38	15.57±2.32**	2.98±0.53	25.80±1.97***~
7-HDPA	7.14±1.61	14.77±5.53	8.45±3.03	10.08±3.35	7.63±1.93	12.43±1.97***
**Bioactive LMs**						
RvT1	1.05±0.47	.140±.077	0.29±0.15	0.98±0.46	0.65±0.35	1.52±0.84
RvT2	1.24±0.71	1.12±0.63	0.48±0.24	0.22±0.14	1.04±0.51	0.12±0.09
RvT3	0.31±0.17	0.31±0.12	0.27±0.14	0.34±0.15	0.22±0.12	0.84±0.42
RvT4	0.14±0.11	0.11±0.07	0.21±0.16	0.45±0.14**	0.18±0.05	0.18±0.07
$RvD1_{n\text{-}3\ DPA}$	28.87±8.97	29.32±14.01	19.92±7.00	33.70±14.25	33.15±21.38	30.81±12.41
$RvD2_{n\text{-}3\ DPA}$	10.75±7.11	13.90±8.35	6.10±3.18	5.01±.230	6.28±2.79	17.81±10.83
$RvD5_{n\text{-}3\ DPA}$	1.10±0.46	0.39±0.30	0.66±0.31	0.43±0.22	0.63±0.26	0.54±0.34
$PD1_{n\text{-}3\ DPA}$	0.53±0.12	0.52±0.26	0.29±0.10	0.32±0.11	0.42±0.12	0.19±0.08~
10S, 17S-diHDPA	0.39±0.18	0.10±0.07	0.04±0.03	0.23±0.18	0.00±0.00	0.03±0.03
$MaR1_{n\text{-}3\ DPA}$	0.19±0.10	0.63±0.25	0.36±0.27	0.28±0.10	0.30±0.11	1.04±0.43
7S,14S-diHDPA	0.18±0.10	0.31±0.16	0.13±0.07	0.14±0.11	0.26±0.17	0.40±0.23
$MaR2_{n\text{-}3\ DPA}$	7.24±1.62	31.33±7.10*	7.43±1.76	42.27±11.94**	9.13±1.83	52.66±13.77***~
**Mediator Families**						
RvT	2.73±0.87	2.93±1.38	1.25±0.34	2.00±0.63	2.08±0.74	2.66±1.17
$RvD_{n\text{-}3\ DPA}$	40.72±9.67	43.60±13.82	24.68±6.90	39.14±14.02	40.07±21.06	49.16±12.77~
$PD_{n\text{-}3\ DPA}$	0.92±0.26	0.62±0.32	0.33±0.11	0.55±0.17	0.42±0.12	0.21±0.09
$MaR_{n\text{-}3\ DPA}$	7.61±1.67	32.38±7.38*	7.93±1.87	42.69±11.90**	9.69±1.89	54.09±14.17***~

Within group

*p<0.05 vs V1

**p<0.05 vs V3

***p<0.05 vs V5

~p<0.05 V1 vs. V6

Table S8. Plasma levels of n-3 DPA metabolome lipid mediators by study visit, PAD subjects.

All values pg/ml plasma Avg±SEM	PAD					
n-3 DPA Metabolome	**V1**	**V2**	**V3**	**V4**	**V5**	**V6**
**n-3 Fatty Acid**						
n-3 DPA	4575.47±1096.68	5771.12±935.01	4800.12±806.64	6846.06±1427.13	3295.67±675.22	10260.10±2602.14***
**Precursors**						
17-HDPA	62.05±11.40^	168.86±31.78*	74.82±16.69	229.00±52.26**	63.90±89.5	400.73±72.79***~
14-HDPA	10.41±1.82	155.43±77.27	60.78±50.00	131.54±36.29	12.35±2.32	208.55±41.09***~
13-HDPA	3.20±0.80	15.99±3.67*	6.10±2.21	25.39±7.68**	3.95±0.81	41.66±10.80***~
7-HDPA	4.07±1.24	25.21±10.38	10.78±4.04	11.91±2.69	8.07±2.69	19.91±4.09~
**Bioactive LMs**						
RvT1	0.19±0.06	0.14±0.10	0.34±0.17	0.71±0.32	0.47±0.20	0.42±0.20
RvT2	0.28±0.14	0.55±0.30	0.70±0.36	0.34±0.25	0.17±0.11	0.47±0.44
RvT3	0.17±0.10	0.15±0.10	0.01±0.01	0.16±0.07	0.24±0.09	0.43±0.29
RvT4	0.01±0.01	0.25±0.11*	0.13±0.10	0.37±0.11	0.13±0.08	0.30±0.11~
$RvD1_{n\text{-}3\ DPA}$	2.52±1.18^	3.49±1.19	5.37±2.89	4.37±1.82	1.38±0.48	8.58±3.14
$RvD2_{n\text{-}3\ DPA}$	1.64±0.86	0.57±0.25	5.32±4.47	1.32±0.50	2.77±2.49	0.18±0.18
$RvD5_{n\text{-}3\ DPA}$	2.79±1.07	1.22±0.58	2.11±0.92	20.3±1.11	1.02±0.60	2.34±1.25
$PD1_{n\text{-}3\ DPA}$	0.22±0.12	0.23±0.13	0.13±0.08	0.09±0.07	0.32±0.17	0.38±0.17
10S, 17S-diHDPA	0.16±0.08	0.18±0.13	0.22±0.12	0.24±0.10	0.31±0.15	0.22±0.11
$MaR1_{n\text{-}3\ DPA}$	0.49±0.22	0.10±0.10	0.12±0.08	0.42±0.20	0.34±0.21	0.43±0.26
7S,14S-diHDPA	0.37±0.26	0.25±0.17	0.22±0.13	0.55±0.22	0.20±0.13	0.29±0.15
$MaR2_{n\text{-}3\ DPA}$	9.07±1.81	52.81±18.76	12.83±2.25	82.23±18.70**	12.07±1.61	136.16±37.35***~
**Mediator Families**	**Mediator Families**					
RvT	0.65±0.22^	1.10±0.37	1.18±0.50	1.58±0.40	1.01±0.22	1.62±0.69
$RvD_{n\text{-}3\ DPA}$	6.96±1.69^	5.28±1.24	12.80±7.42	7.73±161	5.17±2.23	11.11±3.37
$PD_{n\text{-}3\ DPA}$	0.37±0.18	0.41±0.16	0.35±0.16	0.33±0.16	0.63±0.27	0.60±0.17
$MaR_{n\text{-}3\ DPA}$	9.93±1.80	53.16±18.72	13.16±2.27	83.20±18.74**	12.61±1.77	136.87±37.44***~

Within group

*p<0.05 vs V1

**p<0.05 vs V3

***p<0.05 vs V5

~p<0.05 V1 vs. V6

Between Groups

^p<0.05 vs Healthy at V1

Table S9. Plasma levels of EPA metabolome lipid mediators by study visit, healthy subjects.

All values pg/ml plasma Avg±SEM	Healthy					
EPA Metabolome	**V1**	**V2**	**V3**	**V4**	**V5**	**V6**
**n-3 Fatty Acid**						
EPA	2861.58±602.60	3590.65±422.37	2117.15±532.14	5124.18±775.78**	3276.06±817.18	7272.15±1307.15***~
**Precursors**						
18-HEPE	24.03±4.34	213.11±60.99*	32.89±6.04	344.39±74.69**	37.96±7.39	665.46±151.31***~
15-HEPE	15.32±3.26	44.49±10.79*	17.26±4.37	52.35±10.26**	21.33±3.58	105.88±22.79***~
5-HEPE	34.94±9.40	89.24±17.07*	30.15±7.33	97.91±170.06**	56.28±20.61	223.99±44.86***~
**Bioactive LMs**						
RvE1	6.76±2.80	4.89±1.86	4.68±1.53	5.77±2.15	4.35±1.87	4.32±1.25
RvE2	0.97±.042	0.50±0.16	0.23±0.07	0.18±0.08	0.19±0.06	1.00±0.29***
RvE3	0.89±0.24	0.91±0.26	0.67±0.30	0.53±0.30	0.59±0.21	0.35±0.19
**Mediator Families**						
RvE	8.62±3.07	6.30±2.05	5.57±1.62	6.48±2.05	5.13±1.94	5.66±1.17

Within group

*p<0.05 vs V1

**p<0.05 vs V3

***p<0.05 vs V5

~p<0.05 V1 vs. V6

Table S10. Plasma levels of EPA metabolome lipid mediators by study visit, PAD subjects.

All values pg/ml plasma Avg±SEM	PAD					
EPA Metabolome	**V1**	**V2**	**V3**	**V4**	**V5**	**V6**
**n-3 Fatty Acid**						
EPA	2888.09±1136.65	4999.16±957.66*	2855.80±724.96	6491.75±1178.58**	2973.39±792.23	11422.77±2294.95***~
**Precursors**						
18-HEPE	51.58±17.78	203.96±70.77*	41.65±13.22	697.97±213.39**	46.41±11.47	1130.16±346.52***~
15-HEPE	45.97±10.94^	93.66±20.37*	49.05±13.82	149.86±35.37**	39.89±8.65	249.50±56.96***~
5-HEPE	46.03±17.15	124.60±35.92*	50.26±19.31	209.47±62.28**	55.04±16.98	405.38±104.10***~
**Bioactive LMs**						
RvE1	4.10±1.45	8.98±3.59	7.80±2.94	6.13±1.95	4.40±2.11	4.26±2.16
RvE2	0.26±0.13	0.89±0.32	0.42±0.15	1.50±1.12	0.65±0.29	1.17±0.61
RvE3	6.04±5.63	4.53±3.71	4.04±2.68	12.81±10.01	5.66±3.04	10.43±8.07
**Mediator Families**						
RvE	10.39±5.39	14.40±4.65	12.26±4.65	20.44±10.45	10.71±3.15	15.85±7.76

Within group

*p<0.05 vs V1

**p<0.05 vs V3

***p<0.05 vs V5

~p<0.05 V1 vs. V6

Between Groups

^p<0.05 vs Healthy at V1

Table S11. Plasma levels of AA metabolome lipid mediators by study visit, healthy subjects.

**All values pg/ml plasma Avg±SEM**	**Healthy**					
AA Metabolome	**V1**	**V2**	**V3**	**V4**	**V5**	**V6**
**n-6 Fatty Acid**						
AA	17300.73±3981.51	13978.71±1991.19	14490.25±3204.00	17778.31±2762.00	16839.13±2546.33	17790.35±3981.52
**Precursors**						
15-HETE	100.71±19.16	109.48±25.44	105.94±26.20	103.15±22.64	104.91±16.70	101.05±21.39
5-HETE	39.37±9.30	65.00±9.86	27.62±3.72	50.34±5.21**	33.84±5.11	82.84±9.15***~
**Bioactive LMs**						
LXA_4	0.22±0.05	0.20±0.06	0.16±0.06	0.13±0.06	0.19±0.10	0.12±0.60
LXB_4	28.01±8.53	32.40±7.96	26.53±6.20	15.86±3.47	22.55±7.00	37.71±8.89
5S,15S-diHETE	0.80±.026	0.35±0.111	0.84±0.14	0.78±0.20	0.55±0.16	0.50±0.19
15-epi-LXA_4	0.03±0.02	0.20±0.08	0.05±0.03	0.20±0.09	0.03±0.02	0.64±0.24***~
15-epi-LXB_4	1.76±0.82	1.22±0.37	1.15±0.40	0.59±0.24	1.26±0.47	1.23±0.46
13,14-dehydro-15-oxo-LXA_4	0.03±0.02	0.20±0.08	0.05±0.03	0.20±0.09	0.03±0.02	0.64±0.24***~
15-oxo-LXA_4	0.25±0.12	0.12±0.07	0.09±0.04	0.42±0.21	0.12±0.03	0.45±0.15
LTB_4	0.47±0.07	0.91±0.24	0.45±0.07	0.98±0.18**	0.56±0.16	1.45±0.34***~
5S,12S-diHETE	0.35±0.26	0.64±0.19	0.12±0.04	1.08±0.30**	0.11±0.05	1.82±0.44***~
6-trans-LTB_4	0.31±0.10	0.34±0.10	0.12±0.04	0.31±0.09	0.14±0.04	0.81±0.17***~
6-trans-12-epi-LTB_4	0.18±0.14	0.19±0.06	0.20±0.07	0.33±0.09	0.12±0.04	0.24±0.07
20-OH-LTB_4	0.05±0.03	0.06±0.03	0.06±0.02	0.04±0.02	0.08±0.04	0.09±0.04
20-COOH-LTB_4	0.29±0.16	0.34±0.15	0.35±0.17	0.39±0.17	0.85±0.49	0.33±0.27
LTC_4	0.19±0.19	0.13±0.13	0.00±0.00	0.30±0.30	0.00±0.00	0.00±0.00
LTD_4	0.34±0.23	0.10±0.10	0.46±0.34	0.13±0.13	0.45±0.45	0.17±0.12
LTE_4	1.19±0.48	1.23±0.51	0.66±0.36	1.17±0.60	0.97±0.47	0.72±0.50
PGE_2	0.21±0.08	0.35±0.15	0.18±0.06	0.27±0.08	0.40±0.15	0.47±0.19
PGD_2	0.10±0.07	0.40±0.28	0.28±0.21	0.56±0.34	0.28±0.20	0.40±0.21
$PGF_{2\alpha}$	1.20±0.22	0.83±0.27	1.25±0.33	0.74±0.15	1.01±0.24	2.13±0.76
TxB_2	2.09±1.69	2.44±1.43	1.19±0.56	1.70±1.35	1.87±1.01	0.93±0.63
**Mediator Families**						
LX	31.10±8.32	34.69±7.77	28.87±6.08	18.18±3.42	24.73±7.00	41.30±9.03
LT	1.64±0.48	2.50±0.36	1.30±0.15	3.12±0.61**	1.86±0.54	4.73±0.98***~
CysLT	1.73±0.67	1.45±0.52	1.13±0.55	1.61±0.88	1.43±0.66	0.90±0.48
PG	1.51±0.29	1.58±0.34	1.71±0.51	1.57±0.39	1.69±0.41	3.00±0.91
Tx	2.09±1.69	2.44±1.43	1.19±0.56	1.70±1.35	1.87±1.01	0.93±0.63

Within group : *p<0.05 vs V1; **p<0.05 vs V3; ***p<0.05 vs V5; ~p<0.05 V1 vs. V6.

Table S12. Plasma levels of AA metabolome lipid mediators by study visit, PAD subjects.

All values pg/ml plasma Avg±SEM	PAD					
AA Metabolome	V1	V2	V3	V4	V5	V6
**n-6 Fatty Acid**						
AA	23267.79±6370.19	21235.96±3242.58	23564.59±4292.34	25178.53±3049.51	18119.33±1679.24	29599.02±5437.17***
**Precursors**						
15-HETE	256.20±66.08^	243.46±52.80	244.49±56.17	238.19±43.59	191.41±35.91	261.94±51.14
5-HETE	37.30±5.61	58.91±10.16*	41.42±6.60	77.37±23.20	32.46±4.20	112.22±25.41***~
**Bioactive LMs**						
LXA_4	0.15±0.06	0.81±0.61	0.64±0.40	0.33±0.17	0.31±0.19	0.42±0.19~
LXB_4	50.20±14.48	209.86±77.46*	109.61±49.84	100.94±39.86	88.67±28.99	95.21±42.24
5S,15S-diHETE	0.19±0.07^	0.18±0.10	0.59±0.33	0.81±0.27	0.24±0.14	0.37±0.25
15-epi-LXA_4	0.05±0.03	0.24±0.09*	0.03±0.02	0.40±0.17	0.13±0.05	0.93±0.53
15-epi-LXB_4	0.69±0.43	1.30±1.07	1.13±0.28	1.66±0.47	0.84±0.29	1.44±0.52
13,14-dehydro-15-oxo-LXA_4	0.05±0.03	0.24±0.09*	0.03±0.02	0.40±0.17	0.13±0.05	0.93±0.53
15-oxo-LXA_4	0.28±0.08	0.11±0.06	0.33±0.15	0.58±0.20	0.39±0.14	1.58±0.31***~
LTB_4	0.58±0.13	1.08±0.22	0.58±0.26	1.79±0.26**	0.57±0.11	2.85±0.49***~
5S,12S-diHETE	0.18±0.08	0.42±0.14	0.29±0.13	1.29±0.25**	0.04±0.04	2.77±0.71***~
6-trans-LTB_4	0.15±0.06	0.42±0.21	0.39±0.13	0.43±0.23	0.06±0.04	0.43±0.23
6-trans-12-epi-LTB_4	0.25±0.06	0.21±0.13	0.35±0.13	0.24±0.08	0.10±0.04	0.27±0.08
20-OH-LTB_4	0.03±0.02	0.15±0.05*	0.09±0.04	0.03±0.02	0.07±0.04	0.00±0.00
20-COOH-LTB_4	0.15±0.05	0.25±0.08	0.17±0.06	0.21±0.04	0.16±0.09	0.16±0.09
LTC_4	0.00±0.00	0.12±0.012	0.00±0.00	0.41±0.41	0.00±0.00	0.00±0.00
LTD_4	1.22±0.51	0.35±0.24	0.71±0.39	1.93±0.80	2.49±0.86	2.01±0.96
LTE_4	5.94±2.47	4.22±1.39	3.70±0.92	4.39±1.23	22.34±17.93	24.73±19.57
PGE_2	1.04±0.47	11.83±9.16	0.75±0.22	3.29±1.80	0.93±0.35	4.55±4.10
PGD_2	1.26±0.61	5.49±4.19	1.93±1.10	2.12±1.05	1.61±1.01	2.03±1.33
$PGF_{2\alpha}$	2.72±2.02	1.95±0.52	0.98±0.23	1.86±0.59	1.19±0.50	1.12±0.76
TxB_2	7.78±7.68	236.64±230.33	6.81±3.97	5.55±3.83	0.00±0.00	18.03±17.96
**Mediator Families**						
LX	51.63±14.48	212.74±77.39*	112.36±48.84	105.13±40.12	90.72±29.06	100.89±43.18
LT	1.34±0.20	2.53±0.42*	1.88±0.60	3.99±0.55**	0.99±0.18	6.48±1.29***~
CysLT	7.16±2.54^	4.69±1.37	4.41±0.88	6.73±1.57**	24.83±17.62	26.74±19.60
PG	5.03±2.51	19.27±13.77	3.66±1.32	7.27±2.87	3.73±1.05	7.71±5.54
Tx	7.78±7.68	236.64±230.33	6.81±3.97	5.55±3.83	0.00±0.00	18.03±17.96

Within group : *p<0.05 vs V1; **p<0.05 vs V3; ***p<0.05 vs V5; ~p<0.05 V1 vs. V6. **Between Groups:** ^p<0.05 vs Healthy at V1

Table S13. Plasma total SPM and lipid mediator ratios for each study visit, healthy and PAD subjects.

Avg±SEM<br>Healthy Subjects	V1	V2	V3	V4	V5	V6
**Total SPM**	151.61±16.09	188.14±47.86	101.81±7.51	158.88±27.01	154.95±27.46	197.59±30.22
**SPM:PG Ratio**	155.78±12.35	212.337±74.37	105.04±36.20	76.242±15.94	212.73±74.57	149.70±73.28
**O3:AA Ratio**	1.14±0.08	1.83±0.14*	1.21±0.07	2.02±0.20**	1.35±0.13	2.73±0.28***~
**SPM:LTB4 Ratio**	11.07±2.87	10.24±3.41	5.55±0.96	17.48±4.64**	16.85±6.33	11.44±4.85
**RvE1:LTB4 Ratio**	0.62±0.32	0.25±0.08	0.28±0.1	0.74±0.27	0.32±0.12	0.31±0.14
**MaR1:LTB4 Ratio**	0.14±0.08	0.20±0.1	0.24±0.09	0.37±0.15	0.26±0.13	0.14±0.06
**PAD Subjects**						
**Total SPM**	148.53±22.47	335.02±74.79*	244.21±49.60	288.37±47.37	195.18±42.58	350.19±53.81~
**SPM:PG Ratio**	61.45±10.30^	64.21±10.67	141.64±51.00	81.45±20.34	79.72±19.47	415.40±190.05
**O3:AA Ratio**	0.97±0.13	1.55±0.16*	1.03±0.10	1.72±0.20**	1.14±0.13	2.19±0.27***
**SPM:LTB4 Ratio**	3.8±1.2^	3.32±1.04	4.28±1.27	4.5±0.98	3.44±0.72	16.3±6.6
**RvE1:LTB4 Ratio**	0.09±0.04	0.05±0.01	0.10±0.03	0.08±0.02	0.10±0.05	0.06±0.02
**MaR1:LTB4 Ratio**	0.04±0.02	0.03±0.02	0.12±0.06	0.20±0.11	0.18±0.15	0.31±0.16

Within group

*p<0.05 vs V1

**p<0.05 vs V3

***p<0.05 vs V5

~p<0.05 V1 vs. V6

Between Groups

^p<0.05 vs Healthy at V1

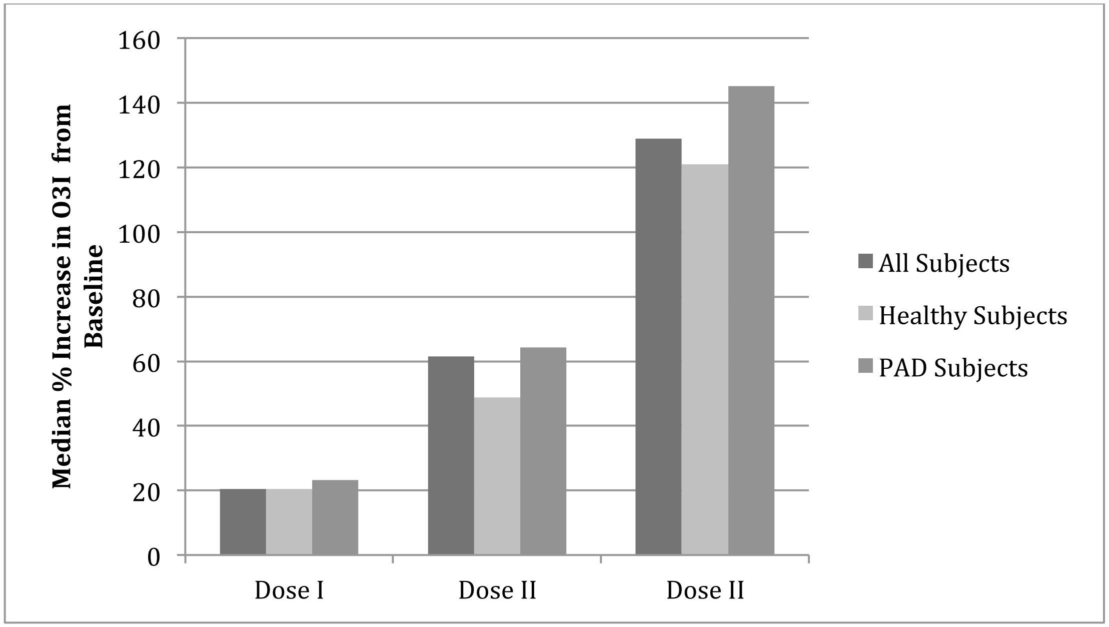

Figure S1. Median percent increase in the Omega-3 Index (the % RBC content of EPA and DHA) for each dose compared to baseline.

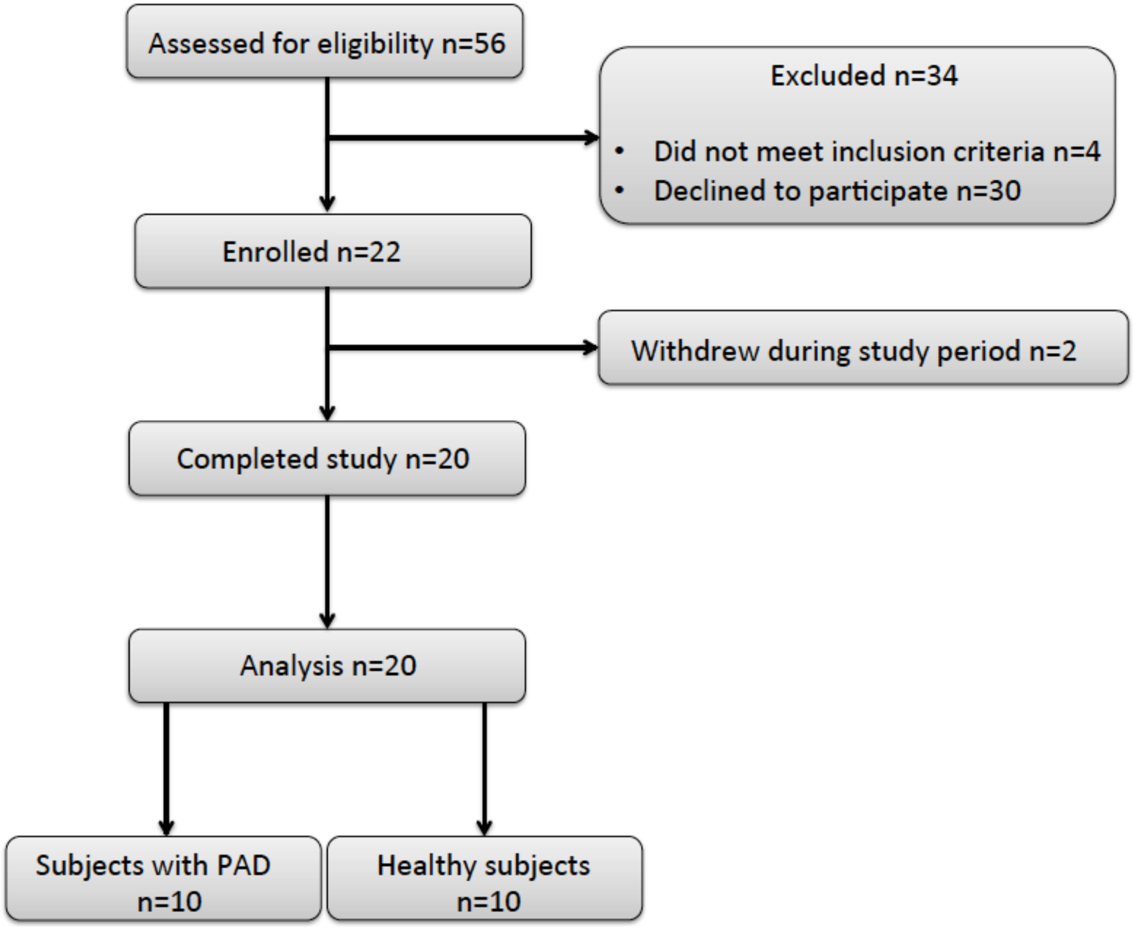

Figure S2. Study recruitment and enrollment schema.
